# Inflammation and atherosclerosis: signaling pathways and therapeutic intervention

**DOI:** 10.1038/s41392-022-00955-7

**Published:** 2022-04-22

**Authors:** Peng Kong, Zi-Yang Cui, Xiao-Fu Huang, Dan-Dan Zhang, Rui-Juan Guo, Mei Han

**Affiliations:** Department of Biochemistry and Molecular Biology, College of Basic Medicine, Key Laboratory of Medical Biotechnology of Hebei Province, Key Laboratory of Neural and Vascular Biology of Ministry of Education, Hebei Medical University, Shijiazhuang, 050017 PR China

**Keywords:** Molecular medicine, Molecular biology

## Abstract

Atherosclerosis is a chronic inflammatory vascular disease driven by traditional and nontraditional risk factors. Genome-wide association combined with clonal lineage tracing and clinical trials have demonstrated that innate and adaptive immune responses can promote or quell atherosclerosis. Several signaling pathways, that are associated with the inflammatory response, have been implicated within atherosclerosis such as NLRP3 inflammasome, toll-like receptors, proprotein convertase subtilisin/kexin type 9, Notch and Wnt signaling pathways, which are of importance for atherosclerosis development and regression. Targeting inflammatory pathways, especially the NLRP3 inflammasome pathway and its regulated inflammatory cytokine interleukin-1β, could represent an attractive new route for the treatment of atherosclerotic diseases. Herein, we summarize the knowledge on cellular participants and key inflammatory signaling pathways in atherosclerosis, and discuss the preclinical studies targeting these key pathways for atherosclerosis, the clinical trials that are going to target some of these processes, and the effects of quelling inflammation and atherosclerosis in the clinic.

## Introduction

Atherosclerosis is the process of plaque formation including various cells, lipids, and debris tissue in the vascular intima,^1^ which is identified as a chronic vascular inflammation mediated by traditional and nontraditional risk factors.^2^ Atherosclerosis was traditionally regarded as a disease of cholesterol accumulation caused by the retention of lipoproteins including low-density lipoprotein (LDL) in the intimal of arteries. LDL taken up by scavenger receptor induces the continuous immune cell infiltration into the atherosclerotic plaque.^3–6^ The hypothesis that atherosclerosis is an inflammatory disease was firstly suggested by Russell Ross in 1999,^7^ based on observations that circulating monocytes infiltrate into the developing fatty streak. The antigens involved in inflammation initiation in atherosclerosis are only recently beginning to be elucidated. Genome-wide association combined with clonal lineage tracing and clinical trials have identified that the mechanisms of innate and adaptive immunes can promote or quell atherosclerosis.^8^ Much evidence suggests that potential major antigens involved in atherosclerosis include neoepitopes generated by oxidized LDL (oxLDL) formed in the vessel wall or when cells undergo apoptotic death.^9^ In addition, other potential antigens released from apoptotic cells in the plaques can further promote the progression of the atherosclerotic plaque, and impaired apoptotic cell clearance can sustain atherogenesis.^10^ Dysregulation of immune cells in the plaques has been recently uncovered by using single-cell transcriptomic and proteomic analyses.^11^ The plaques in symptomatic patients exhibited the characterization of a distinct CD4^+^ T-cell subset and T cells to be activated and differentiated, whereas in the plaques from asymptomatic patients, T cells and macrophages were also activated and raised interleukin-1β (IL-1β) signaling. Altogether these observations underscore the diversity of phenotype and functions of immune cells in atherosclerotic plaques and the interplay between systemic immune response and local event at the plaque site acts as drivers of plaque instability.

New evidence suggests that remnants of triglyceride-rich lipoproteins promote the development of atherogenesis, highlighted by deleterious effects of apolipoprotein (Apo) CIII.^12^ Based on the intimate relationship between lipids and inflammation, a metabolic-immune hypothesis of atherosclerosis has recently been proposed aiming to provide a complementary view regarding the effect of lipids and inflammation on the pathogenesis of atherosclerosis.^13^ Therefore, inflammation can drive vascular hyperplasia without traditional cardiovascular risk factors and involves aspects of plaque biology that lead to the complications of advanced atherosclerosis.

Given the relationship between inflammation and atherosclerosis, treatment of atherosclerosis from an inflammatory perspective appears to be a more effective anti-atherosclerotic modality. Although no direct evidence supports that selectively intervention of inflammation can improve outcomes in atherosclerosis patients,^14^ clinical trials have unequivocally shown that modulation of inflammation can forestall atherosclerosis and its complications,^15,16^ which represents the transformation of inflammation in atherosclerosis from theory to practice.^14^ Therefore, a gamut of attractive therapeutic strategies for modulation of inflammation has been suggested in the treatment of atherosclerosis, including inhibiting pro-inflammatory cytokines, blocking key inflammatory signaling pathways, and promoting inflammatory resolution.^2^ In addition, new immunotherapies for atherosclerotic cardiovascular events could be identified by clarifying the dysregulation of specific immune within the plaque regions, beyond the traditional management of cardiovascular risk factors and the use of standard lipid-lowering agents.^11^

In this review, we summarize the knowledge on cellular participants and inflammatory signaling pathways in atherosclerosis, and discuss these pathways as potential therapeutic targets for atherosclerosis and clinical trials that are doing targeting some of these processes, and the effects of quelling inflammation and atherosclerosis in the clinic. Much of our understanding of the roles of pivotal inflammatory signaling pathways in atherosclerosis is based on findings from the most recent experimental studies and clinical trials. Although both suppressing inflammation and promoting resolution may lead to unwanted effects, taming inflammation might substantially offer multiple benefits for human health.^2^

## Cells involved in atherosclerosis

**Fig. 1:**
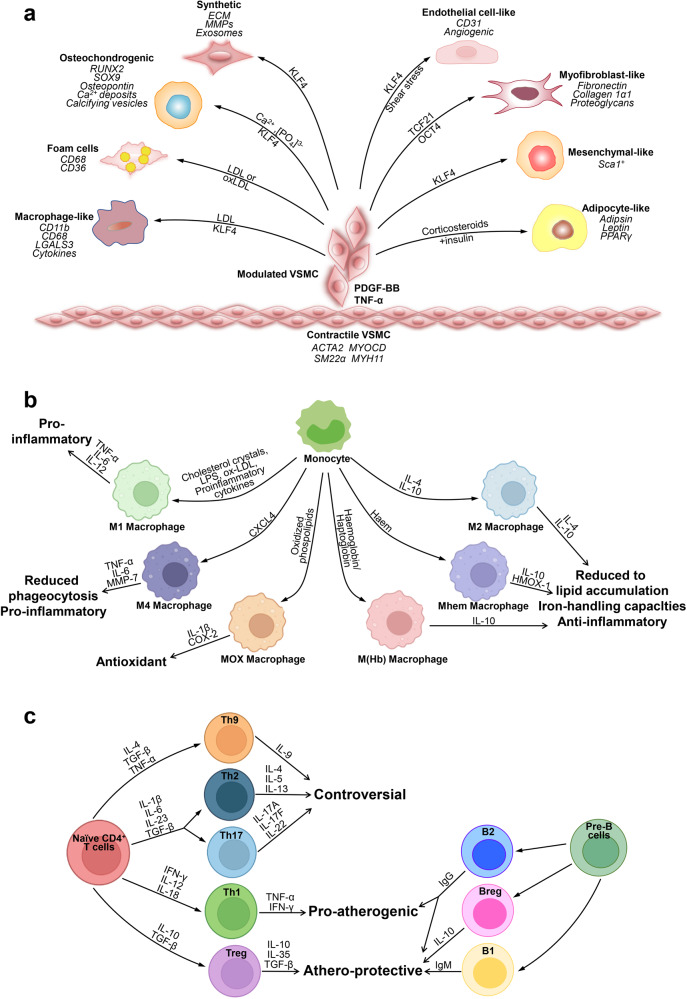
Key cells involved in atherosclerosis. **a** Phenotypic switching of VSMCs in atherosclerosis. In the healthy arterial wall, VSMCs are a contractile phenotype expressing contractile proteins (ACTA2, SM22α, Myocardin (MYOCD), and MYH11). Upon PDGF-BB and TNF-α, VSMCs switch to a synthetic phenotype, which increases the production of ECM, exosomes, pro-inflammatory cytokines, and MMPs. VSMCs release calcifying vesicles to propagate vascular calcification. KLF4 promotes phenotypic switching of VSMCs into foam-like, macrophage-like, osteochondrocyte-like, adipocyte-like, and Sca1^+^ mesenchymal-like VSMCs. Shear stress induces transdifferentiation of VSMCs into endothelial-like cells. The transcription factors TCF21 and OCT4 (octamer binding transcription factor) promote modulation of VSMCs into atheroprotective myofibroblast-like phenotype. **b** Plasticity and function of macrophages in atherosclerosis. Monocytes differentiate toward various phenotypes of macrophages in response to stimuli in atherosclerotic lesions. Among them, M1 macrophages secret pro-inflammatory cytokines; M2, M (Hb), and Mhem phenotypes are anti-inflammatory; Mox macrophages exhibit an antioxidant effect; and M4 phenotypes express pro-inflammatory cytokines and have impaired phagocytosis. **c** Lymphocytes in atherosclerosis. CD4^+^ T cells can differentiate into distinct lineages including T helper 1 (Th1), Th2, Th17, T regulatory (Treg), and many other Th cells. Th1 cells produce TNF-α and IFN-γ, indicating a pro-inflammatory and pro-atherogenic role of Th1 cells. Treg cells promote inflammatory resolution and dampen atherosclerosis progression via the production of IL-10 and TGFβ. The effect of Th2, Th9, and Th17 cells on the development of atherosclerosis remains controversial. B cells can exert both a pathogenic and protective role in atherosclerosis. B cells have two main subsets B1 and B2 cells. B1 cells exert an atheroprotective effect by the release of IgM antibodies against oxidation-specific epitopes. Similarly, Breg cells also act atheroprotective by the production of IL-10. B2 cells exhibit both pro-atherogenic and atheroprotective depending on the inflammatory microenvironment

Atherosclerotic plaques are complex structures that consist of vascular cells and immune cells. In this section, we discuss how various types of cells contribute to vascular inflammation and key cell types involved in atherosclerosis and key roles (Fig. 1).

### Vascular cells

#### Vascular endothelial cells

Endothelial barrier integrity plays a key role in maintaining a fluid balance between the circulation and tissues and vascular homeostasis. Studies indicate an association between endothelial dysfunction, subsequent elevation in the levels of endothelial factors, and development and intensification of coronary artery disease and atherosclerosis.^17^ As secretory cells, endothelial cells (ECs) exert significant paracrine and endocrine actions through their influence on the underlying VSMCs or on circulating blood elements, such as platelets and white blood cells. They produce and release a variety of vasoactive substances, such as endothelin-1 (ET-1), nitric oxide (NO), prostacyclin, angiotensin 2 (Ang II), vascular cell adhesion molecule 1 (VCAM-1), intercellular adhesion molecule 1 (ICAM-1), as well as vascular endothelial growth factor (VEGF) and platelet-derived growth factor (PDGF), which regulate vasodilation, platelet aggregation, and monocyte infiltration.^18^ The mitochondrial ubiquitin ligase MARCH5, a critical regulator of mitochondrial dynamics, mitophagy, and apoptosis, is demonstrated to protect endothelial function against hypoxia/ischemic injury via serine/threonine kinase AKT/eNOS (endothelial NO synthase) axis.^19^ Inhibition of receptor-type vascular endothelial protein tyrosine phosphatase (VE-PTP) elicits phosphorylation of eNOS to break endothelial dysfunction.^20^ β-catenin facilitates endothelial survival by promoting activation of eNOS and expression of the flow-dependent anti-apoptotic gene.^21^ The beneficial effect of NO is partially mediated by inhibiting the expression of MCP-1 (monocyte chemoattractant protein-1).^22^ Thus, regulation of endothelial integrity and function is necessary for maintaining vascular homeostasis and for preventing the development of atherosclerosis.

#### Vascular smooth muscle cells

VSMCs are a major type of cells presented at all stages of atherosclerotic development. Substantial heterogeneity of VSMCs in morphology and gene expression related to atherosclerosis has been identified by transcriptional profiling of healthy arteries, including observation of atypical, rare VSMCs with Sca1 positive and VSMCs with expressing genes related to phenotypic switching, suggesting that there are VSMC subtypes relevant to a particular disease.^23,24^ Phenotypically modified VSMCs can produce a large number of extracellular matrix (ECM) proteins (such as elastic fibers, collagens, proteoglycans, and MMPs), and secrete a wide range of cytokines (MCP-1, IL-1β, and IL-6) that can regulate the function of neighboring cells in a paracrine manner, and release various extracellular vesicles (EVs) to induce vascular calcification.^25,26^

VSMCs play both beneficial and detrimental roles in the development of atherogenic plaques, which depends on the nature of their phenotypic changes. Besides synthetic phenotype, VSMCs adopt another phenotype, such as pro-inflammatory macrophage-like phenotype,^23,27^ mesenchymal stem cell-like phenotype,^23^ fibromyocyte phenotype,^24^ osteogenic phenotype,^28^ EC-like,^29^ adipocyte-like,^30^ and intermediate cell phenotype^31^ during the development of atherosclerotic disease (Fig. 1a). Although VSMCs can return to the contractile state under some conditions,^31,32^ to date, no evidence shows that VSMCs in the intima can relocalize in the media. The wide differences of VSMC phenotypes between medial, intimal, and plaque could influence the ultimate role of VSMCs in atherosclerosis.^33^

**Fig. 2:**
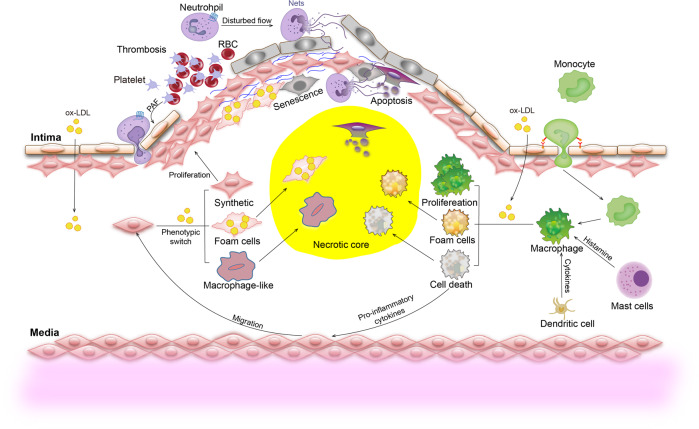
Overview of inflammatory responses in atherosclerotic development. At the early stages, activated platelets mediate firm adhesion between platelets, leukocytes, and the vascular endothelium by secreting platelet-activating factor (PAF). In progressing plaques, VSMCs migrate from the medial to the subendothelial space where they undergo proliferation and developing fibrous cap. OxLDL induces phenotype switching of VSMCs to synthetic, macrophage-like VSMCs and the formation of foam cells. Excessive deposition of lipids triggers VSMC apoptosis and senescence, leading to necrosis. At predilection sites with the disturbed flow, neutrophil-released neutrophil extracellular traps (NETs) induce desquamation of endothelial cells and lead to rapid occlusion of the affected vessels. Monocyte-derived macrophages ingest oxLDL and release pro-inflammatory cytokines. Excessive deposition of lipids, as well as cytokines and histamine released from dendritic cells and mast cells, trigger the proliferation, polarization of macrophages, or even cell death

Although the prevailing point is that VSMCs exert beneficial roles in advanced atherosclerosis as they can stabilize fibrous cap and are major cells producing ECM, single-cell RNA-sequencing combination with lineage tracing have revealed much more-diverse phenotypes of VSMCs in the various stages of atherosclerosis.^1,2^ Lipid uptake alters the VSMC phenotype into macrophage-like and foam-like.^34^ The detrimental effects of these macrophage-like VSMCs have been supported by the specific deletion of KLF4 (transcription factor krueppel-like factor 4) in VSMCs.^35^ The macrophage-like or foam-like VSMCs are pro-inflammatory and contribute to plaque vulnerability (Fig. 2).

Smooth muscle 22 alpha (SM22α), an important cytoskeleton-associated protein, is required for maintaining the contractile phenotype of VSMCs and is a sensitive and specific marker for identifying the dedifferentiation and phenotypic switching of VSMCs.^36–38^ Recent studies from us and others have indicated that SM22α binds and regulates the organization of actin cytoskeleton,^38,39^ and maintains Ang II-activated extracellular signal-regulated kinase (ERK) 1/2 contraction signaling by mediating mitogen-activated protein kinase (MAPK) phosphatase 3 ubiquitination degradation, and thus modulating the VSMCs phenotype in the physiological state.^40^ In addition, SM22α also acts as an adapter or scaffold protein to modulate the formation and translocation of signaling complexes, and to maintain adaptive phenotype alteration of VSMCs. The arteries of *Sm22α*^−/−^ mice develop enhanced inflammatory response and oxidative stress, which contributes to neointimal hyperplasia through different signaling mechanisms,^41–43^ suggesting that *Sm22α*^−/−^ VSMCs have transited to a synthetic or pro-inflammatory state.^44^ A more recent study revealed that SM22α inhibits abdominal aortic aneurysm formation by preventing VSMC phenotypic switching by suppressing ROS/nuclear factor (NF)-κB.^45^ These findings support the notion that molecular changes by SM22α loss may have already initiated the early inflammation of atherosclerosis.^46^

Transcriptome profiling reveals an increased tendency to develop atherosclerosis in SM22α-deficient mice.^46^ SM22α mediates suppression of VSMC proliferation and neointima hyperplasia via blockade of Ras-ERK1/2 pathway^47^ and inhibits migration via uncoupling Ras-Arp2/3 interaction in synthetic VSMCs.^48^ Phosphorylation of SM22α facilitates Ang II-induced ROS production via activating protein kinase Cδ (PKCδ)-p47phox axis in actin dynamics-dependent manner, contributing to hypertrophy and hyperplasia of VSMCs in vitro and in vivo.^49^ We also demonstrated that insulin-independent glucose transporter type 4 (GLUT4) translocation in proliferative VSMCs involves SM22α.^50^ Furthermore, TNF receptor-associated factor 6 (TRAF6)-mediated SM22α ubiquitination promotes activation of glucose-6-phosphate dehydrogenase (G6PD) and reduces the production of nicotinamide adenine dinucleotide phosphate, leading to impaired glutathione (GSH) homeostasis and VSMC survival.^51^ In addition, we have indicated that deficiency of SM22α enhances the interaction between VSMCs and macrophages and triggers VSMC apoptosis via promoting zinc finger protein-36 (ZFP36)-mediated decay of Bcl-2 mRNA.^44^ More recently, we elucidated the roles of SM22α in nuclear receptor LXRα (liver X receptor α)-modulated cholesterol homeostasis of VSMCs.^52^ Loss of SM22α blocked LXRα nuclear import and reduced ATP-binding cassette transporter (ABCA1)-driven cholesterol efflux via promoting F-actin depolymerization, which aggravated the development of atherosclerosis. Despite protecting against vascular inflammation and oxidative stress, accumulation of SM22α protein accelerates senescence of VSMCs and vascular aging via suppression of murine double minute 2 (Mdm2)-mediated degradation of p53 in vitro and in vivo.^53^ Collectively, SM22α is involved in various VSMC behaviors via regulating different signaling pathways and plays important roles in the pathogenesis of atherosclerosis.^54^

#### Fibroblasts

Fibroblasts in the adventitia are metabolically active cells and play a prominent role in the development of atherosclerosis.^55^ Latest studies have indicated that the adventitia provides a dynamic microenvironment for the regulation of both structural and functional properties of all three arterial layers. Importantly, resident adventitial fibroblasts may be the first cells in the vascular wall in response to inflammatory and environmental stimuli.^56^ Activated fibroblasts enhance interaction with ECs and VSMCs and regulate their functions, as well as recruit immune cells into the vessel wall.^43^ The most important roles of fibroblasts in advanced atherosclerosis involve modulation of the inflammatory response and ECM protein production, and maintenance of the structural integrity of the plaque and balance of MMP production, to promote beneficial tissue remodeling, alongside preventing plaque rupture.^55^ Regulation of fibroblast activities to control or reverse the progression of atherosclerosis may be an attractive target for therapeutic intervention.

#### Platelets

Platelets are central to inflammation-related manifestations of atherosclerosis. Beyond effects on the immune cell infiltration, platelets regulate the metabolism of cholesterol by modifying, binding and endocytosing LDL via scavenger receptors and promoting the formation of macrophage foam cells.^57^ The platelet-activating factor (PAF) released from platelets can induce integrins to mediate firm adhesion between the endothelium and leukocytes or platelets (Fig. 2).^58^ Platelet-derived extracellular vesicles (PEV) produced by activated platelets can trigger the initiation of atherosclerosis.^59^ Ablation of platelet apoptosis can reduce atherosclerosis in diabetes mice, leading to a more stable plaque by preventing platelet-monocyte interactions and subsequently monocyte activation.^60^ The current advances in anti-inflammatory therapies have identified an inflammatory mediator effect of thrombosis secondary to platelet activation.^61^

### Immune cells

Atherosclerosis is multifacetedly triggered by contributions of the immune system in the circulation and at the local vascular lesions. Single-cell proteomics combined with transcriptomic analyses revealed specific characteristics of immune cell dysregulation within the plaques that lead to clinical ischemic stroke or myocardial infarction.^11^

#### Monocytes/macrophages

Macrophages in plaques are not exclusively derived from monocytes, but mainly rely on their own local proliferation.^62^ Macrophages exert atheroprotective effects under homeostatic conditions, through both the clearance of lipoprotein by endocytosis and apoptotic cells by phagocytosis or efferocytosis, thereby abolishing the inflammatory processes involved in plaque formation.^63^ Macrophages, as a major source of chemokines, cytokines, matrix protein-degrading enzymes, play a critical role in sustained local inflammatory responses and plaque rupture.^64^ Apoptotic cell-derived nucleotides activate the proliferation of efferocytosis macrophages, which is essential for inflammation resolution and atherosclerosis regression, which may be new ways to treat non-resolving inflammatory diseases.^65^

Macrophages can be categorized into M1 (classically activated) macrophages that contribute to tissue destruction and secrete pro-inflammatory factors and M2 (alternatively activated) macrophages that produce anti-inflammatory factors. Distinct macrophage subsets resolved at the single-cell transcriptional level revealed activated and pro-inflammatory functional phenotypes, which is not accurately defined by the M1 and M2 phenotypes.^11^ Furthermore, additional plaque-specific macrophage phenotypes are recently identified, including Mhem, Mox, and M4 (Fig. 1b). Hemorrhage-residing Mhem macrophages participate in hemoglobin clearance via phagocytosis of erythrocyte and exhibit increased cholesterol efflux, which is a subset of atheroprotective and resistant to foam cell formation,^66^ as they highly express the cholesterol transporters ABCA1 and ABCG1 and the nuclear receptors, LXR-α and LXR-β. Mox macrophages, a pro-atherogenic subset induced by oxidized phospholipids, protect from oxidative stress. M4 macrophages displayed reduced phagocytic capacity, increased neutrophil recruitment, and more effective neutrophil extracellular trap (NET) induction,^67^ which represents an atherogenic phenotype. In addition, iron accumulation in the plaques may induce the M (Hb) macrophage differentiation, which expresses CD163 (scavenger receptor cysteine-rich type-1 protein M130) and has, therefore, protective properties in atherosclerosis. Furthermore, a novel transcriptional intermediary state between nonpolarized (M0) and inflammatory M1-like macrophages were identified. These macrophages were characterized by the high expression of GATA2, a hematopoietic transcription factor, leading to impaired efferocytosis and efferosome maturation during the earliest stages of atherosclerotic development in humans.^68^

Collectively, the functions and phenotypes of macrophages can vary widely based on several factors, including the macrophage origin, the stage of atherosclerosis, and the microenvironment^69^ (Fig. 2). Such determinants not only confer the appearance and functionality of macrophages but also define their heterogeneity at a single-cell level, suggesting that different subsets coexist within one tissue.^2^ Given the key role of macrophages in atherosclerotic initiation, progression, and resolution, nanoparticle (NP)-based imaging modalities specifically targeting macrophages, as novel diagnostic and therapeutic strategies, are used to therapeutically manipulate macrophages in the plaques to increase plaque stability and to reduce the risk of cardiovascular disease.^70,71^ For example, a small interfering RNA (siRNA) NP targeting macrophage Ca^2+^/calmodulin-dependent protein kinase has been shown to improve all signs of plaque stability in advanced atherosclerosis of Ldlr^−/−^ mice^72^ via an increase in expression of MER tyrosine-protein kinase to drive inflammation resolution.^73^

#### Lymphocytes

Increased number of lymphocytes is well identified as an independent risk factor for atherosclerotic disease,^74^ and reduced lymphocyte number is also considered to be associated with increased risk,^75^ suggesting the complex role of the immune system in the development of atherosclerosis.^76^

T cells play a critical role in cellular immunity, including CD4^+^, CD8^+^, natural killer (NK) T cells, and helper T cells etc. Most cytokines are secreted by T cells. CD4^+^ T, termed as T helper (Th) cells, are a heterogeneous group and can differentiate into Th1, Th2, Th17, Treg, and many other Th cells.^77^ Th1 cells release TNF-α and IFN-γ, exerting a pro-atherogenic role.^78^ Th2 cells that are a lineage of CD4^+^ T cells, primarily interact with B cells and produce IL-4, −5, and −13. Based on both pro- and anti-atherogenic actions of Th2 cells, the effect of these cells on atherosclerosis appears to be more complex compared with Th1 cells.^79^ Th9 cells can produce IL-9 upon transforming growth factor (TGF)-β and IL-4 stimulation. However, it is unknown that Th9 cells are pro-atherogenic or anti-atherogenic.^80^ Th17 cells release IL-17A, F, and IL-22, which are pro-inflammatory. Despite protecting against fungal and bacterial infections,^81^ however, the role of Th17 cells in atherosclerotic diseases remains controversial as both pro-atherogenic^82,83^ and anti-atherogenic actions^84^ have been reported.

There are very low numbers in regulatory T cells (Tregs) in the plaques, compared with other chronic inflammatory tissues,^85^ representing an increased local inflammation in the plaques. The anti-inflammatory cytokines TGF-β, IL-10, and IL-35 are important for Treg cell suppression.^86^ Adoptive transfer of chemokine (C-X3-C motif) receptor-1 (CX3CR1) transduced-Treg cells improve homing to the plaques and dampen progression of atherosclerosis.^87^ Recently identified Treg cell-related biomarkers in deteriorated atherosclerosis were used to distinguish patients with myocardial infarction from those with stable coronary disease.^88^ Recently, developments in high-parametric cell immunophenotyping by single-cell RNA-sequencing, mass cytometry, combined tools exploring antigen-specificity reveal that pathogenic ApoB-reactive T cells evolved from immunosuppressive and atheroprotective CD4^+^ Treg cells and lose their protective properties over time.^89^

The CD8^+^ T cell in human and mouse atherosclerosis revealed activated, cytotoxic, dysfunctional, and exhausted cell phenotypes.^90^ CD8^+^ Treg cells play a protective effect in advanced atherosclerosis through limiting increases in Th1 cells and macrophages.^91^ In addition, adoptively transferred CD8^+^ T cells promote the protective effects of peptide immunization on atherosclerosis in ApoE^−/−^ mice.^92^

Collectively, Th1 cells play pro-inflammatory and pro-atherogenic, whereas the effects of Th2, Th9, and Th17 cells on the development and progression of atherosclerosis remain controversial.^80^ Treg cells suppress the activity of CD4^+^ Th and cytotoxic CD8^+^ T cells and promote the anti-inflammatory phenotype of macrophages and resolution of inflammation (Fig. 1c).^93^ CD4^+^ and CD8^+^ T cells in the plaques from symptomatic patients with recent cardiovascular events were activated, differentiated, and exhausted compared with their blood counterparts.^11^ The dynamic balance between different T-cell subsets controls the formation of vulnerable and obstructive plaques during atherosclerotic development.^94^

B cells can reveal both protective and pathogenic effects in atherosclerosis. B1 and B2 cells are two main subsets identified in human atherosclerotic plaques.^95^ New observations from the effects of the therapies targeting B cells in autoimmune diseases suggest that the distinct B-cell subsets and different immunoglobulins play a prominent role with atherogenic and protective effects, and it is observed that B-cell subset depleting (modifying) therapies may exert the beneficial side effects on atherosclerotic concomitant disease.^96^ Besides the production of antibodies, B cells also play regulatory roles through the production of IL-10 and therefore these cells are named regulatory B cells.^97,98^ Recent studies demonstrate that a low number of IL-10^+^ B cells in atherosclerosis patients is associated with inflammatory condition^99^ and that alarmin-activated B cells accelerate atherosclerosis after myocardial infarction via plasma cell-immunoglobulin dependent mechanisms.^100^ Hence, the identification of human B-cell subsets and their production of IL-10 would help to better understand the role of these cells in atherosclerosis, and to distinguish which of these subsets truly have a pro- or anti-atherogenic role (Fig. 1c).

#### Neutrophils

Neutrophils are the most abundant circulating white blood cells in humans, have received far less notice until recently. Neutrophils localize at sites of plaque erosion,^101^ which correlates negatively with endothelial continuity.^102^ Neutrophil factors attract and activate macrophages. In response to disease stimuli, neutrophils undergo a specialized series of reactions that eventually lead to NETs formation, a complex structure composed of nuclear chromatin and proteins of nuclear cytoplasmatic and granule origin.^103^ NETs underlie the communication between neutrophils and monocytes directly to stimulate cells into the atherosclerotic plaques and modulate the inflammatory response.^104,105^ Extracellular cholesterol crystals induce neutrophils to release NETs, which trigger macrophages to express a precursor form of pro-IL-1β. The striking correlation between NETs and dying SMCs, necrotic core sizes, and thin fibrous caps observed in the atherosclerotic mice suggests a direct cytotoxic action of NETs (Fig. 2),^106^ which accelerate atherosclerosis during endotoxinemia.^107^

#### Mast cells

Mast cells are migrant cells in connective tissue with many granules rich in histamine and heparin. Accumulated studies have confirmed that mast cells differentiated from hematopoietic stem cells are also critical for atherosclerosis. They are present in the intimal and epicardial plaques of the aorta in patients, and the number increases with the development of atherosclerosis.^108^ Mast cells are amplified by self-cascade, increasing leukocyte infiltration and further increasing plaque area as they were degranulated later.^109^ Protease and Histamine mediators secreted by mast cells mediate apoptosis of ECs, VSMCs, and macrophages (Fig. 2). These cells are rich in trypsin-like enzymes that degrade ApoE and ApoA1, reduce intracellular cholesterol efflux, promote the transforming of macrophages and VSMCs into foam cells, and promote the development of atherosclerosis eventually.^110^

#### NK cells

NK cells play an immunoregulatory role in the pathogenesis of atherosclerosis.^111^ CD160, a unique activating NK cell receptor, can mediate cytolytic responses and production of cytokines.^112^ Symptomatic plaques of carotid atherosclerosis are associated with increased NK cell infiltration and higher levels of serum NK-activating receptor ligands.^113^ However, there is still no consensus on whether the role of NK cells in atherosclerosis is related to their cytolytic activity or rather to cytokine secretion. Initial studies showed that depletion of NK cells resulted in decreased atherosclerosis. A recent study demonstrated that hyperresponsiveness or genetic depletion of NK cells do not affect development of atherosclerosis.^114^ As a whole, the effects of NK cells on the development of atherosclerotic plaques began to be concerned about recently, and however, the conclusions seem to be conflicting and need to be further investigated.^115^

#### Dendritic cells

Dendritic cells (DCs) have multiple roles in the development of atherosclerosis in both direct and indirect manners. DCs uptake lipids and form foam cells, contributing to an atherosclerotic plaque at the early stage. Furthermore, the mature DCs can promote activation and proliferation of T cells via presenting antigens to T cells and clear apoptotic cells by efferocytosis. In addition, DCs also regulate the activity of other immune cells by production of cytokines. For example, DCs can recruit circulating haematopoietic cells or leukocytes to the injured vascular sites via secreting chemokines.^116^ The conditioning cytokines produced by DCs control the differentiation of T cells into various T effectors (Th1, Th2, Th17, and Treg), regulate the activation of B cells and cytotoxic T cells, and polarization of macrophages, which ultimately lead to immune destruction of vascular regions and the onset of atherosclerosis. The study in Ldlr^−/−^ mice found that Atg16l1-deficient CD11b^+^ DCs produced a TGF-β-dependent tolerance phenotype and promoted CD4^+^ Treg cell expansion, reducing the development of atherosclerosis.^117^ Recent studies have reported that Alisol B 23-acetate (23B), a new promoter for cholesterol efflux in DCs, alleviates inflammation and dyslipidemia in advanced atherosclerosis of mice, thereby controlling the atherosclerotic inflammatory state.^118^ These findings expand our understanding of how DCs affect atherosclerosis and provide new potential approaches to prevent atherosclerosis.

## Signaling pathways in atherosclerosis

**Fig. 3:**
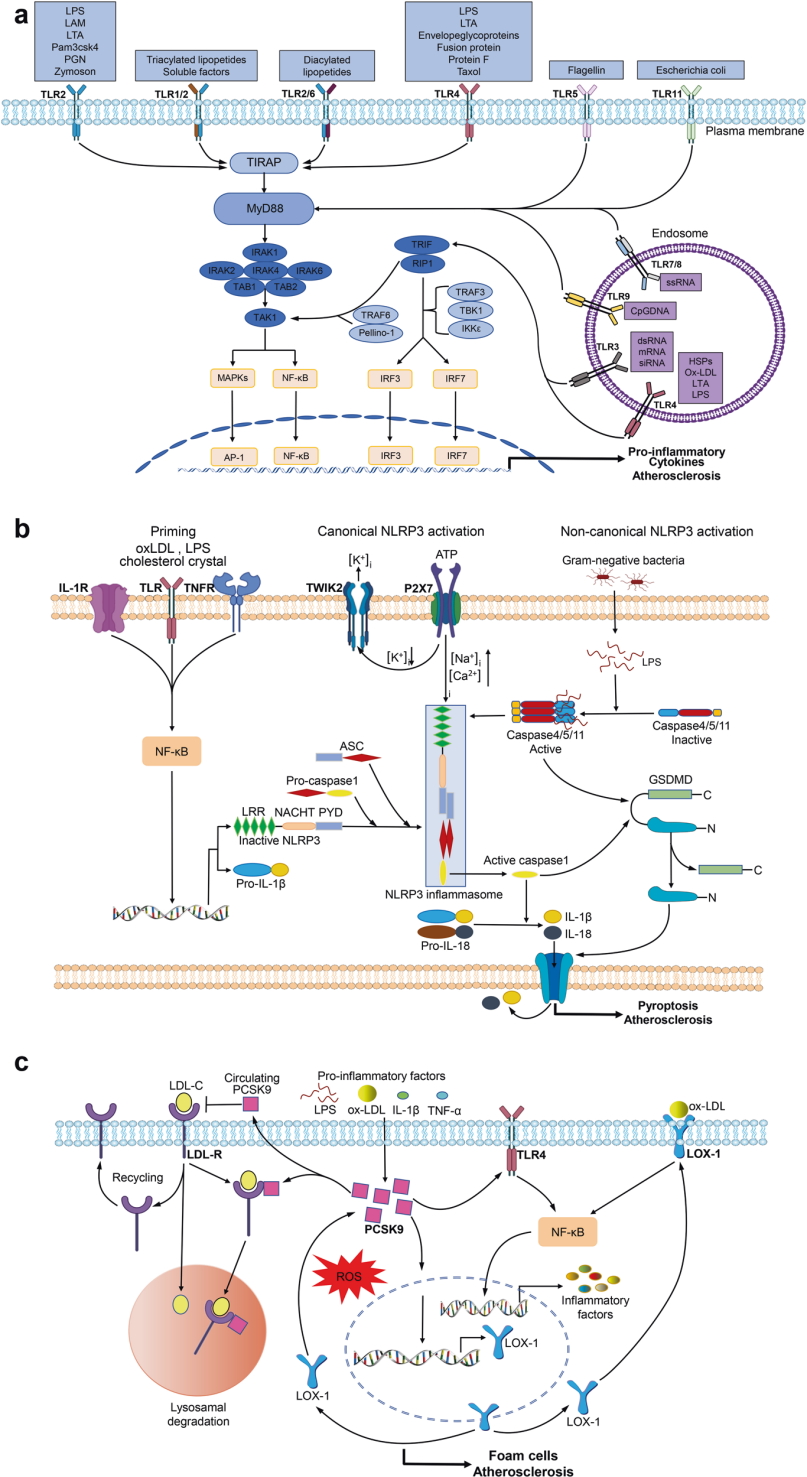
Key signaling pathways in atherosclerosis. **a** TLR signaling pathway. TLR stimulation triggers MyD88 to interact with IRAK4 (interleukin-1 receptor-associated kinase 4), which transmits signals into NF-κB and MAPK to activate the expression of inflammatory cytokines via the MyD88-dependent pathway. Endosomal TLRs transmit signals through the TRIF-dependent pathway. TRIF together with RIP1, TRAF6, and Pellino-1 activate TAK1 or with noncanonical TBK1 (Tank-binding-kinase 1) and IKKε (IKKi) activates interferon regulatory factor 3 (IRF3) and 7 to induce inflammatory cytokines. **b** NLRP3 inflammasome pathways. The activation of NLRP3 inflammasome has two steps: the priming and the activation. The priming is triggered by endogenous cytokines or microbial molecules. The activation of NLRP3 inflammasome includes canonical and noncanonical pathways. The canonical activation induces caspase-1 activation, which processes pro-IL-1β/pro-IL-18 to IL-1β/IL-18 active form. The noncanonical activation induced by mouse caspase-11, and human caspase-4 and caspase-5, indirectly promotes the expression of pro-IL-1β/pro-IL-18. Activated caspase-1 and caspase-11 can cleave GSDMD (gasdermin D), leading to the formation of pores in the plasma membrane and causing pyroptosis and the release of IL-1β/IL-18. **c** PCSK9 pathways. The expression of PCSK9 can be activated by oxLDL, LPS, and pro-inflammatory cytokines in ECs, VSMCs, and macrophages. In the absence of PCSK9, the LDL–LDLR complex is internalized. Subsequently, internalized LDLR-LDL-C complex dissociates and LDLR is recycled to the cell surface, whereas LDL-C is directed to lysosomes for degradation. PCSK9 can mediate internalized LDLR-LDL-C complex to degrade, and promote oxLDL-induced inflammation through increasing expression of LOX-1 and TLR4, which increases oxLDL uptake and upregulates inflammatory cytokine expression via activation of ROS and NF-κB

The signaling pathways mediated by immune, inflammatory mediators are implicated within the atherosclerotic lesion. Understanding these processes helps researchers to create a range of novel biomarkers and treatment modalities. Herein, we mainly give the idea about the important signaling pathways involved in atherosclerosis (Fig. 3), and discuss the recent preclinical studies targeting some of these processes.

### Toll-like receptors

The toll-like receptors (TLRs) are a class of transmembrane proteins that include a cytoplasmic region homologous to the IL-1 receptor and an extracellular leucine-rich domain. Although TLRs are extensively expressed in immune cells such as monocytes, macrophages, DCs, neutrophils, lymphocytes, and vascular cells such as fibroblasts, ECs, VSMCs, the specific TLRs in each cell type play a unique role in the immune response.^119^

#### Classification and function of TLRs

TLR signaling induces the production of anti-microbial peptides, pro-inflammatory cytokines, adhesion molecules, reactive nitrogen, and oxygen species. In mammals, a total of 13 TLRs have been identified to date, among which TLR1-10 functions in humans and TLR12-13 in mice,^120^ and each TLR has the specificity for different ligands to a certain degree.^121^ For example, heterodimerized TLR2/TLR1 binds to triacylated lipopeptides derived from mycoplasma and Gram-negative bacteria, and diacylated lipopeptides from Gram-positive bacteria are recognized by TLR2/TLR6 heterodimers. TLR3 can recognize siRNA, double-stranded RNA, and self RNA released by injured cells. TLR4 recognizes exogenous and endogenous ligands, including oxLDL, heparan sulfate, HSPs, fibrinogen, hyaluronan fragments, beta-defensin, lipoteichoic acid (LTA), lipopolysaccharide (LPS), protein F, and envelope glycoprotein, that are released at chronic inflammation sites in response to tissue stress or damage, such as atherosclerosis (Fig. 3a).^122^

#### TLR signaling pathways

TLR signaling pathways are classified as a MyD88 (myeloid differentiation primary response protein 88)-dependent pathway to trigger NF-κB activation and a TRIF (TIR-domain-containing adaptor protein inducing IFNβ)-dependent pathway. MyD88 is required for all TLRs (except TLR3) and members of IL-1 receptor family, and activates NF-κB and MAPK signaling pathways and inflammatory cytokine expression. TLR activation triggers the interaction of MyD88 with interleukin-1 receptor-associated kinase 4, which is the most upstream serine/threonine kinase of the complex.^123^ In addition to the classical MyD88-dependent pathway, endosomal TLRs can also transmit signals through the TRIF-dependent pathway. TRIF with TRAF6 (TNF receptor-associated factor 6), TRADD (TNFR1-associated death domain protein), RIP1, and Pellino-1 forms a multiprotein signaling complex that activates NF-κB, MAPK, or TAK1 pathways. Furthermore, TRIF recruits signaling complexes of noncanonical TBK1 (tank-binding-kinase 1) and IKKi (IKKε) to induce the phosphorylation of IRF3 (interferon regulatory factor 3) and IRF7, which translocated into the nucleus to induce the expression of type I IFNs (Fig. 3a).^124^

#### Role of TLRs in atherosclerosis

Dysregulation of TLRs is a key mechanism for inflammation and atherosclerosis, contributing to the development of cardiovascular diseases.^125,126^ Current human, animal, and epidemiological experiments demonstrate that chronic infectious diseases and related biological pathogens are involved in the progression of atherosclerosis. Accumulating evidence suggests that the expression of TLR4 and TLR2 increases in peripheral blood mononuclear cells, monocytes, ECs, and VSMCs in atherosclerotic disease. The agonists of TLRs are more likely to be endogenous factors. Highly immunogenic oxLDLs are associated with the upregulation of TLRs.^127^ Oxidized phospholipids in minimally modified LDL can bind and activate TLR4 on macrophages.^128^ Recent study revealed that oxLDL regulated the expression of TLR2 and TLR4 in cultured HUVECs.^129^ Furthermore, studies in mice have determined a central role for TLRs 1-9 in the development of atherosclerosis.^130–134^ Activation of TLR2 and TLR4 may play a profound role in infection-related atherosclerosis.^135^ TLR4 deficiency improved atherosclerosis in ApoE knockout (ApoE^−/−^) mice and LDL receptor-deficient (Ldlr^−/−^) mice,^136,137^ and markedly reduced atherosclerosis induced by oral bacteria. Subsequent follow-up research also confirmed that deletion of the shared TLRs signaling^135^ or adaptor including MyD88, TRAM, and TRIF leads to a reduction in plaque burden to varying degrees.^138–140^ However, it is controversial about the functions of TLR7 and TLR9 in the formation of atherosclerotic plaques.^141^ The hypothesis that pathogen-mediated TLR activation contributes to atherosclerosis remains to be demonstrated by more research.

#### Potential therapeutic targets

The roles of TLRs in inflammation promote the development of the therapeutic potential of targeting TLRs in the treatment of atherosclerosis. Animal experiments suggest that inhibiting TLR signals or blocking pathogen-associated molecular patterns (PAMPs) may be used for the treatment or prevention of atherosclerosis.^142,143^ Drugs both inhibiting the activity of intestinal bacterial LPS and blocking the TLR2- and 4-dependent signaling pathways can reduce the inflammation-activating pathways in atherosclerosis of mice and humans.^144,145^ Infection of *Porphyromonas* *gingivalis* accelerated atherosclerosis, which can be prevented via immunization in animal models.^146^ Ldlr^−/−^ mice immunized with *Streptococcus pneumoniae* display an increase in the specific antibodies to oxLDL and decreased atherosclerotic lesion.^147^ However, multiple clinical trials revealed that anti-infective therapies are inefficacy in mitigating atherosclerotic diseases.^148^ Increasing evidence suggests that annual influenza vaccination reduces all-cause mortality of atherosclerotic patients, with no negative impact on recipients.^135^ Immunization with oxLDL or natural homologous LDL by vaccine formulations containing different adjuvants exerts atherosclerotic protection in animals.^149^ Therefore, vaccines against exogenous and endogenous antigens may represent a major translational goal for the treatment of atherosclerosis.

### NLRP3 inflammasome

Inflammasomes are the complexes of multimeric cytosolic proteins and assemble in response to damage-associated molecular patterns and PAMPs, representing the inflammatory responses.^150^ The NLRP3 inflammasome (LRR, NACHT, and PYD domains-containing protein 3) that is well known, senses the endogenous danger signal activated by cholesterol crystals, activates caspase-1 to cleave pro-IL-1β and pro-IL-18 into mature and biologically active IL-1β and IL-18.^122,151^ The NLRP3 inflammasome is highly expressed in a variety of cell types, including innate immune cells and non-immune cells involved in the pathogenesis of the atherosclerotic cardiovascular disease.^152–154^

#### Structure of NLRP3 inflammasome

NLRP3, as a cytosolic protein, consists of an amino-terminal PYD (pyrin domain) that interacts with ASC (apoptosis-associated speck-like protein containing a caspase recruitment domain), a central NACHT domain (nucleotide-binding and oligomerization domain) that possesses ATPase activity, and a rare LRR (leucine-rich repeat) domain that can fold back onto the NACHT domain to induces autorepression.^155^ ASC, as an adapter protein, provides a bridge between NLRP3 and caspase-1. The PYD domain is also required for ASC self-association as well as interaction with NLRP3.^156^ Caspase-1 is initially synthesized as inactive zymogens and is produced by proteolytic cleavage.^157^

#### Activation of NLRP3 inflammasome

Canonical activation of NLRP3 inflammasome involves two hits: priming and activation. The priming step is induced by TLRs and cytokine receptors, such as TNF receptor or IL-1 receptor, and then NF-κB is activated and upregulates transcriptionally NLRP3 and pro-IL-1β, which promotes NLRP3 to form inflammasome assembly.^158,159^ The second step requires the activated NLRP3 oligomerization to recruit caspase-1 via ASC adaptor, ultimately leading to proteolytic cleavage of pro-IL-1β and pro-IL-18 into their active forms.^122^ Priming by oxLDL depends on binding of oxLDL to CD36 and the formation of CD36-TLR4-TLR6 complex, and internalization of the oxLDL results in lysosomal damage via activation of NLRP3.^160^ The P2X7 receptors are ligand-gated ion channels and can be opened by binding of extracellular ATP, which increases Na^+^ and Ca2^+^ influx and promote K^+^ efflux through coordinating with the K^+^ channel, leading to the activation of NLRP3 inflammasome.^161^

The noncanonical NLRP3 activation pathway is initiated by these caspases direct binding to intracellular LPS (iLPS) produced by Gram-negative bacteria, independent of TLR4, the conventional LPS receptor.^162^ For example, caspase-4, -5, and -11 can indirectly promote the mature pro-IL-1β and pro-IL-18 by activation of the NLRP3 inflammasome via binding to iLPS.^163,164^ Moreover, activated caspase-4, -5, and -11 induce pyroptosis via cleaving gasdermin D in a similar manner as caspase-1,^165,166^ and do not process directly pro-IL-1β and pro-IL-18.^167^ Caspase-11 induces production of extracellular ATP and in turn activates P2X7 receptor and promotes K^+^ efflux, leading to the activation of NLRP3 inflammasome and release of IL-1β,^168^ suggesting the link between noncanonical and canonical NLRP3 inflammasome pathways (Fig. 3b).^169^ In addition, the NLRP3 inflammasome is also activated by LPS via an alternative pathway that does not require K^+^ efflux, ASC speckle formation, or pyroptosis, which was only found in human monocytes.^170^

#### Regulation of NLRP3 inflammasome

The activation of NLRP3 inflammasome is tightly regulated by several innate immune molecules during infection and inflammation.^171^ The basal expression of NLRP3 is not typically sufficient for NLRP3 inflammasome activation. Numerous regulators promoting the NLRP3 inflammasome formation and activation have been identified, such as MyD88 and TRIF contributing to the priming of the NLRP3 inflammasome,^172^ caspase-8, and FADD (Fas-associated death domain protein) to mediate the priming and activation of the canonical and noncanonical NLRP3 inflammasome.^173^ In addition, the stress granule protein DDX3X binds to and activates NLRP3 inflammasome.^174^ In contrast, negative regulators of the NLRP3 inflammasome, such as the E3 ligase A20/TNF-α-induced protein 3 and TGF-β-activated kinase 1 (TAK1), prevent its excessive activation and suppress inflammation.^175,176^

In addition, the NLRP3 inflammasome is also regulated by cellular processes, such as ribosome stalling, translation inhibition,^177^ and post-translational modifications of NLRP3. Recent observation suggested that NEK7, a serine/threonine kinase, is required for activation of NLRP3 inflammasome via interaction with the nucleotide-binding domain and LRR of NLRP3.^178^ The post-translational modifications are critical for the priming of NLRP3 inflammasome activation. However, some of the post-translational modifications prevent NLRP3 inflammasome activation,^169^ or stabilize the NLRP3 in a signal-competent but the auto-suppressed state.^179^ The ubiquitination of NLRP3 mediated by E3 ligases and deubiquitinating enzymes regulate NLRP3 stability. E3 ubiquitin ligases that promote K-48 linked polyubiquitination, such as tripartite motif-containing 31, attenuate activation of NLRP3 inflammasome by proteasomal degradation.^180^ Additionally, NLRP3 was sumoylated at basal state via conjugating with small ubiquitin-like modifier (SUMO)-2/-3, which was mediated by MAPL/MUL1, a SUMO E3 ligase.^181^ The sumoylation of NLRP3 promotes oligomerization of ASC and activation of the inflammasome. SENP3 (SUMO-specific protease 3) mediates the NLRP3 desumoylation, leading to reduced ASC recruitment and NLRP3 inflammasome activation.^182^ Recent studies revealed the importance of the NLRP3 phosphorylation in the priming step.^183^ JNK1-mediated phosphorylation of NLRP3 at S194 is a key priming event, which is required for the NLRP3 activation. The phosphorylation at Ser295 of NLRP3 promotes NLRP3 oligomeric assembly.

#### Role of NLRP3 inflammasome in atherosclerosis

Previous clinical and experimental studies have demonstrated that IL-1β, as a pro-atherogenic cytokine, is involved in atherosclerosis progression, suggesting that NLRP3 inflammasome is presumably a key element in atherosclerotic pathogenesis.^153,184^ Indeed, the expression of NLRP3 inflammasome is increased in the plaques and peripheral blood mononuclear cells of atherosclerosis patients, which possibly reflected the severity of atherosclerosis. ASC knockout mice displayed reduced neointimal hyperplasia after injury, indicating the linking between the NLRP3 inflammasome and atherogenesis.^185^ Using bone marrow-transplanted mice provide further evidence that NLRP3 inflammasome participates in atherosclerotic progression. Loss of ASC, NLRP3, or IL-1 in bone marrow cells of Ldlr^−/−^ mice ameliorated the atherosclerotic lesion.^186^ Deficiency of NLRP3 in Ldlr^−/−^ mice seem to have merely small influences on atherogenesis.^187^ Other studies also showed that treatment with the selective NLRP3 inhibitor MCC950 or lentivirus-mediated NLRP3 silencing reduced atherosclerotic progression in ApoE^−/−^ mice, further suggesting a causative role of NLRP3 inflammasome.^188^ However, other studies showed that there were no significant differences in macrophage infiltration or plaque size between ApoE^−/−^ mice and ApoE^−/−^ mice with deficient in ASC, NLRP3, or caspase-1, representing conflicting views that atherosclerosis progresses independently of the NLRP3 inflammasome in ApoE^−/−^ mice.^189^ Possible explanations for this discrepancy are differences in the atherogenic diet, the hyperlipidaemia level, and even the sex-specific effects, which may affect host immune and inflammatory responses,^153^ as NLRP3 deficiency in bone marrow cells attenuated atherosclerotic lesion in female but not male Ldlr^−/−^ mice.^190^

Most studies showed that monocytes promote phenotypic switching of VSMCs through activation of NLRP3 inflammasome, which exerted a likely detrimental role in the plaque stability in humans.^154^ In addition, mitochondrial dysfunction, oxidative stress, lysosome rupture, and endoplasmic reticulum (ER) stress as well as extracellular Ca^2+^, which are involved in activation of inflammasome, all existed in the plaques, especially in necrotic cores, and however, few studies have been conducted on these mechanisms in atherosclerosis.^191^

#### Potential therapeutic targets

Because of the pivotal role of NLRP3 inflammasome in the development of atherosclerosis, inhibiting NLRP3 inflammasome activation or the pharmacological inhibitors targeting NLRP3 inflammasome components that include P2X7 receptors antagonist, inhibition of caspase-1, and anti-IL-1, may have beneficial effects in protecting from inflammatory damage in atherosclerosis.^192^ To date, several small-molecule drugs targeting NLRP3 inflammasomes have been identified and employed in preclinical studies of cardiovascular inflammation.^179^ The synthetic small molecules, MCC950, CY-09, and OLT1177 bind directly to the NACHT domain of NLRP3 and block its ATPase activity.^169^ MCC950 that is the most representative inhibitor of the NLRP3 inflammasome activation, potently inhibits ATP-triggered, NLRP3-mediated IL-1β production,^193^ reduces macrophage infiltration and lesion size via attenuating inflammation and pyroptosis in hypercholesterolemia and hyperglycemia-induced atherosclerosis in mice.^188,194,195^ CY-09 directly interacts with the NACHT domain and disrupts ATP binding to NLRP3, which shows excellent preventive and therapeutic effects in mouse models.^196^ The small-molecule inhibitors VX-765, a caspase-1 inhibitor prodrug activated by intracellular esterases, can mitigate atherosclerosis in mice.^197^ For therapeutic purposes, the specificity of the potent target sites would be the critical prerequisite for the development of new inhibitors of NLRP3 inflammasome.

In recent years, NPs have been widely reported for the specific delivery of anti-inflammatory agents, peptides, antibodies, or small RNA directly on atherosclerotic lesions.^70^ For example, systemic delivery of methotrexate to macrophages via nanoconstructs has been shown to constitute an effective strategy for limiting the progression of atherosclerosis.^198^ Anti-miR33 nanotherapies significantly promoted reverse cholesterol transport and notably regulated adaptive immunity via modulating macrophage polarization and Tregs differentiation.^199^ Similar improved approach, applying the novel plug and play functionalized erythrocyte nanoplatform targeted drug delivery and acetic acid-control drug release show a marked improvement in atherosclerosis.^200^ In addition, based on the inherent affinity of macrophages for atherosclerotic lesions to construct biomimetic NPs fabricated with a macrophage membrane coating on the surface of rapamycin-loaded poly copolymer NPs has been demonstrated to effectively inhibit the progression of atherosclerosis.^201^ Although these findings suggest that using NPs to deliver anti-inflammatory substances is promising results, further basic research is necessary to understand more about the underlying functional mechanisms of the NPs. Other therapeutic strategies and current clinical trials targeting the NLRP3 inflammasome for atherosclerosis treatment would be described in the Atherosclerosis Therapies section of this review.

### Proprotein convertase subtilisin/kexin type 9

Proprotein convertase subtilisin/kexin type 9 (PCSK9) is now identified as an important and major player in the pathophysiology of atherosclerosis.^202^ PCSK9 is a soluble protein synthesized as a zymogen, and degrades LDL receptors (LDLR) upon activation by autocatalytic cleavage in the ER and subsequently increases LDL cholesterol (LDL-C) levels.

#### Expression of PCSK9

PCSK9 is mainly synthesized and secreted by the liver, and is also expressed in the central nervous system, lung, kidney, intestine, and blood vessel cells. The expression of PCSK9 is regulated by different transcription factors, including sterol-response element-binding proteins (SREBP) 1 and 2, peroxisome proliferator-activated receptor (PPAR) α and γ, as well as sirtuin (SIRT) 1 and 6. Among them, SREBP1 and SREBP2 have been well understood,^203^ which promote the expression of PCSK9 gene. In addition, PPARγ increases the PCSK9 gene expression in the liver,^204^ while peroxisome proliferator-activated receptor-α (PPARα),^205^ SIRT1, and SIRT6 decrease its expression.^206^ Pro-PCSK9 includes five domains: a signal peptide (aa 1–30), an N-terminal prodomain (aa 31–152) that is cleaved in the ER, a catalytic domain (aa 153–421) that contains the active sites Asp186, His226, and Ser386, a region containing 18 amino acids that links the catalytic domain to the C-terminal cys-his-rich domain (CHRD) (aa 440–692).^207–209^ Post-translational modifications are required for pro-PCSK9 maturation, in particular, autocatalytic cleavage of the prodomain of pro-PCSK9 in the ER. PCSK9 in vascular cells such as ECs, VSMCs, and macrophages can also be activated by pro-inflammatory cytokines and LPS.^210–212^

#### Role of PCSK9 in atherosclerosis

PCSK9 promotes hepatic LDLR lysosomal degradation, which has a pivotal role in cholesterol homeostasis and atherosclerosis. PCSK9 can also regulate LDLR levels in immune and vascular cells.^213^ PCSK9 secreted by VSMC and ECs downregulates LDLR expression on the surface of macrophages^214^ in a paracrine manner,^215^ and inhibits foam cell formation. *Pcsk9*^−/−^ SMCs protected from neointimal hyperplasia via the reduced capacity of proliferation and migration.^216^

Beyond cholesterol metabolism, other physiological processes are also regulated by PCSK9, such as adipogenesis modulation, immune response, and interaction with many other cellular receptors, including oxLDL receptor-1 (LOX-1), VLDL receptor (VLDLR), and ApoE receptor 2 (ApoER2), CD36, and LDL receptor-like protein-1 (LRP-1).^212^ Some of these, including CD36 and LRP-1, are potent signaling receptors expressed on vascular and hematopoietic cells and thus PCSK9 might very well regulate important hemostatic systems, including inflammation, hemostasis, and tissue repair.^217^ PCSK9 in VSMCs exacerbates atherosclerotic lesion via self-reinforcing crosstalk of ROS/NF-κB/LOX-1/oxLDL axis activated by inflammation.^202^ PCSK9 induces LOX-1 expression and in turn, LOX-1 activation upregulates PCSK9 expression in VSMCs, which is a positive feedback regulation between the two.^210^ PCSK9 and LOX-1 share many cellular signaling pathways activating vascular inflammation and cellular apoptosis (Fig. 3c).

PCSK9 promotes the activation of platelet and thrombosis by interaction with platelet CD36.^218^ PCSK9 upregulates TLR4 expression and NF-κB activation to induce pro-inflammatory cytokine and tissue factor expression in a variety of tissues,^219,220^ and thus PCSK9 acts as a pro-inflammatory mediator. Several studies demonstrated that PCSK9 deficiency significantly reduced the levels of plasma pro-inflammatory cytokines IL-6, IL-8, TNF-α, and MCP-1.^221^ In addition, several epidemiological studies evaluated the association between PCSK9 and some key inflammatory markers including fibrinogen, hs-CRP (high-sensitivity C-reaction protein), and white blood cells, further suggesting the link between PCSK9 and inflammation.^212^ Importantly, the possible non-lipid-lowering effects of PCSK9, such as activation of platelets, the inflammatory burden of atherosclerosis, and metabolism of triglyceride-rich lipoprotein, have been identified and inhibition of PCSK9 acts as a safe potential therapeutic target to prevent thrombosis in patients who may be at higher risk because of elevated PCSK9 levels by hereditary or acquired factors as inflammation or using statins.^217^

#### Potential therapeutic targets

During the past decade, passive immunotherapy using PCSK9 monoclonal antibodies (mAbs) is an important breakthrough in lipid-lowering therapy. Various approaches, including mAbs, peptide inhibitors, or silencing PCSK9 mRNA expression and translation, have been identified to reduce the levels of plasma PCSK9.^222–224^ However, the studies of animal models showed that PCSK9 inhibitors offer a direct anti-inflammatory effect independent of the reduction in LDL-C, which needs further research.

Vaccination is an actively pursued new strategy for PCSK9 inhibition. PCSK9 vaccines AT04A and alirocumab decreased levels of serum cholesterol, reduced vascular and systemic inflammation, and limited atherosclerosis development.^225,226^ The PCSK9 vaccine Qβ-003 was recently reported to obviously decrease LDL-cholesterol and total cholesterol (TC) and to reduce the lesion size in ApoE^−/−^ mice. In addition, infiltration of macrophage and expression of TNF-α and MCP-1 were obviously reduced in ApoE^−/−^ mice administered the PCSK9Qβ-003 vaccine.^227^ Furthermore, the PCSK9Qβ-003 significantly increased the plaque stability and regulated cholesterol transport by upregulating the expression of LXR-α and ABCA1.

The specific property of a PCSK9 vaccine is able to induce the host to generate anti-PCSK9 antibodies that can disrupt the interaction between PCSK9 and LDLR.^228^ To this end, L-IFPTA (liposomal immunogenic fused PCSK9-tetanus peptide plus Alum adjuvant) is a recently designed novel PCSK9 vaccine.^229^ L-IFPTA vaccine contains a PCSK9 sequence as a B-cell epitope, and a T-helper cell epitope belonging to tetanus toxin proteins. So far, L-IFPTA vaccine has been shown to have long-lasting preventive and therapeutic effects on atherosclerosis in mice.^230,231^ The L-IFPTA vaccine also decreases the increased IFN-γ-inducing T-cell levels and obviously elevates Th2 cell levels and IL-4 and IL-10 expression in hypercholesterolaemic mice.^228^ Altogether, these studies identify the potential of L-IFPTA vaccine as a potent candidate for the treatment of dyslipidaemia and atherosclerotic disease.^228^ Other therapeutic strategies and current clinical trials targeting PCSK9 for atherosclerosis treatment would be described in the “Atherosclerosis therapies” section of this review.

### Notch

The Notch is an evolutionarily conserved cellular signaling pathway that mediates intercellular communication and is involved in the regulation of most tissue development and homeostasis.

#### Core Notch pathway

Four isoforms of Notch receptors (Notch1–4) and five types of Notch ligands (Dll 1, 3, and 4, and Jagged1 and 2) are expressed in mammals.^232^ A single precursor of Notch receptors that is initially synthesized migrates to the Golgi apparatus and is cleaved by a furin-like protease into a membrane-distal and a membrane-anchored subunit. In the canonical Notch signaling, the Notch ligand binding to its receptor results in the removal of the extracellular fraction, and then two protein hydrolysis by A disintegrin and metalloprotease (ADAM10/17) and subsequently by a γ-secretase, respectively,^233^ results in the generation of the Notch active form, NICD (Notch intracellular domain). The NICD translocates into the nucleus to activate the transcription of target genes via binding to RBPJ (recombination signal-binding protein for immunoglobulin kappa J region) and other factors such as p300 and CBP-associated factor (PCAF). The Notch signaling also functions through “noncanonical” pathways. The activity of NICD occurs independently of the binding to canonical ligand or RBPJ and the γ-secretase cleavage.^234^ Noncanonical Notch signaling can interact with other pathways, including mTORC2 (mammalian target of rapamycin complex 2)/AKT, IκB kinase (IKK) α/β pathways,^235^ or Wnt/β-catenin. For example, noncanonical Notch pathway modulates mitochondrial function to promote cell survival by activation of mTORC2/AKT signaling with PINK1 (PTEN-induced kinase 1).^236^

#### Roles of Notch in atherosclerosis

Accumulating evidence has demonstrated that Notch protects from the dysfunctions of endothelium caused by inflammatory cytokines,^237^ and that Notch receptors mediate communication between ECs and VSMCs, and regulate the cell phenotypes.^238–240^ A growing number of studies show that the Notch plays a critical role in the transduction of the signals induced by flow shear stress to ECs.^241^ Activated Notch provides an anti-inflammatory, anti-atherogenic and pro-survival environment^242,243^ and maintains the endothelial integrity by mediating the formation of the tight junction complex in EC.^244,245^

Notch is also key signaling for regulating the structure and function of VSMCs. The expression of Notch receptors 2 and 3, and the main ligand Jagged1 are found in VSMCs. The mutation of Notch 2 and 3 can lead to defects in the development of VSMCs, which provides strong evidence for the implication of Notch signaling in regulating vascular differentiation and maturation during angiogenesis.^246^ Jagged1-Notch3 signaling mediates nidogen-2 to maintain the contractile phenotype of VSMCs through in vitro and in vivo.^247^ Although abundant studies have identified that Notch has a central role in the control of SMC development and function, and intimal repair, much less is known about its role in the fate of VSMCs in atherosclerotic development and progression. Recent studies demonstrated that Notch signaling is required for the adhesion of VSMCs but not other types of VSMC-derived cells in the formation of the cap in mouse atherosclerosis, and reduction of Notch signaling is a prerequisite for medial VSMC mediating the development of plaque,^248^ suggesting that sequential loss and gain of Notch signaling is required for the recruitment of cap SMC population in atherosclerosis.^249^

Notch regulates atherosclerosis by controlling the differentiation of macrophages into M1 or M2 subtypes.^235^ Notch1 induces M1 differentiation and enhances inflammatory responses by promoting MCP-1, IL-6, and TNF-α secretion. Instead, inhibition of Notch1 promotes differentiation of M2 macrophage and the production of the anti-inflammatory cytokines IL-1 receptor antagonist (IL-1Ra) and IL-10.^250,251^ It was shown that treatment with the DAPT, a Notch inhibitor, reduced macrophage migration and suppressed ICAM-1 expression in macrophages, thereby reducing macrophage infiltration in the plaques of ApoE^−/−^ mice.^252^ Another study demonstrated that DAPT enhanced the anti-atherogenic activity of the LXR ligand agonist T317 in ApoE^−/−^ mice while limiting the development of hypertriglyceridemia and fatty liver.^253^ In addition, Notch1 upregulates IL-6 expression in activated macrophages via the NF-κB pathway.^254^ IL-6 activates STAT3 (the signal transducer and activator of transcription-3), which in turn induces the expression of DLL1 and activates Notch signaling, establishing a positive feedback loop.^255^ DLL4-Notch1 axis promotes the polarization of M1 macrophages and blocks M2 polarization in the development of atherosclerosis. Overall, these studies suggest that Notch signaling could act as a target in different cell types to interfere with atherosclerosis progression. Therefore, further research is needed to better understand the variety and contradictory actions of Notch signaling, which may help in ameliorating and preventing atherosclerotic development.

#### Potential therapeutic targets

Increasing evidence suggests the aberrant regulation of Notch signaling in inflammatory diseases, and thus the ligands and receptors of Notch are expected to act as attractive therapeutic targets. In theory, the potential of targeting Notch signaling to modulate inflammation, as a new approach, is attractive, and however, their design and implementation are difficult, at least partly due to the broad involvement of Notch signaling in homeostatic and regenerative processes.^256^ The two main strategies targeting Notch have been identified, including GSIs (γ-secretase inhibitors) that can block the active NCID release to reduce the levels of active Notch, and mAbs that can either inhibit ligation upon cell contact or prevent proteolytic cleavage via stabilizing the NRR-region. GSIs have been shown to reduce atherosclerosis progression in ApoE^−/−^ mice, which exert reduced ICAM-1 expression and total plaque areas.^252^

Compared with inhibition of pan-Notch, the use of mAbs is a more attractive approach, as mAbs are beneficial over conventional drugs in terms of specificity for the target, potency, and dosing frequency. IgG1 is the most perfect molecular scaffold to design mAb, as it has a long plasma half-life and the ability to mediate the function of a potent effector.^257^ However, The inherent traits of IgG1 structure must be considered for Notch-targeting. The mAb binding to effectors may lead to not only therapeutic efficacy but the also detrimental effect to tissue expressing Notch, which depends on both mAb target and indication.^256^ Animal studies show that mAbs targeting individual Notch ligands or receptors exert reduced intestinal toxicity, compared with that found by GSI treatment.^258,259^ Additional experimental data show the potential of targeting Notch pathway in atherosclerosis treatment. Blockade of DLL4-Notch signaling with an anti-DLL4 mAb decreased accumulation of macrophage, diminished calcification of plaque, reduced insulin resistance, and inhibited progression of atherosclerosis in Ldlr^−/−^ mice.^260^ Since the Notch signaling is a potentially attractive target candidate in atherosclerosis, further research is needed to elucidate the distinct biological roles of Notch receptors and ligands in the plaque progression and to validate the individual potentials as novel therapeutic strategies.^256^

### Wnt

The Wnt pathway participates in all different stages of atherosclerosis, from endothelial dysfunction to lipid deposit, and from initial inflammation to plaque formation.

#### Wnt family of proteins

Wnt proteins are a family of secreted lipid-modified glycoproteins. There are 19 different Wnt proteins that have been identified in humans. The proteins of the Frizzled family are the best characterized Wnt receptors,^261^ including 10 Frizzled isoforms in humans and animals. The Frizzled proteins, as unconventional G-protein-coupled receptors, contain a cysteine-rich extracellular domain (CRD), a seven-transmembrane spanning domain, and a cytoplasmic tail.^262^ Wnt ligands bind to the Frizzled receptors via interaction with the Frizzled CRD.^263^

#### Wnt signaling pathways

Wnt pathway builds a complex signaling regulatory network. Three intracellular pathways for Wnt signaling have been identified, which include the canonical or Wnt/β-catenin pathway that activates gene transcription through β-catenin, and the noncanonical Wnt/PCP (planar cell polarity) pathway that regulates cytoskeletal dynamics through activating JNK (C-Jun N-terminal kinase) by small G proteins; and Ca^2+^-dependent pathways (Wnt/Ca^2+^ pathway), which affects cellular adhesion and related gene expression through the release of intracellular Ca^2+^.^264^ The canonical Wnt/β-catenin signaling mediates the post-translational regulation of β-catenin that is a chief downstream effector. The β-catenin is degraded by multiprotein complex β-catenin destruction complex in the absence of Wnt ligands. During intracellular signaling, Wnt ligands bind to frizzled receptors and LRP 5 or 6 that are members of the LDLR gene family, leading to β-catenin translocating into the nucleus to trigger the transcription of target genes. In canonical Wnt signaling, Wnt ligands bind to several different receptors to promote a variety of cellular processes, depending on the presence of coreceptors and the formation of the receptor complexes.^265^ In addition, Wnt signaling is regulated by DKK (dickkopf) and Wnt ligand scavenging sFRP (secreted frizzled receptor protein) family.

#### Role of Wnt signaling in atherosclerosis

Beyond regulating cell proliferation and differentiation, the Wnt pathway also controls lipid homeostasis and storage.^266^ The activation of Wnt signaling is negatively correlated with the atherosclerotic severity. Involvement of Wnt in atherosclerosis was initially found in clinical patients carrying the Wnt co-receptor LRP6 mutation.^266^ These patients show increased LDL-C, triglycerides, and fasting glucose levels.^267,268^ Genetic experiments in mice suggest that the function loss of LRP6 is associated with coronary artery disease.^267^ Similarly, LRP5 also prevents atherosclerosis. Reduced serum levels of Wnt ligands are directly involved in the development of atherosclerotic disease.^269^

It has recently been shown that with lipid-lowering potentiates, activation of the Wnt pathway enhances IL-4 responsiveness in macrophages through the PGE2/STAT3 axis.^270^ Dickkopf-2 (DKK2), a negative regulator of Wnt/β-catenin, is involved in the activation of macrophages during atherosclerosis. Knockdown of DKK2 significantly reduces the expression levels of genes associated with M1-type macrophage polarization but upregulates markers of M2-type macrophage polarization and significantly attenuated foam cell formation. DKK2 knockdown activates the Wnt/β-catenin signaling by promoting the entry of β-catenin into the nucleus of macrophages, which leads to macrophage inactivation.^271^ Meanwhile, the Wnt/β-catenin signaling pathway was activated and DKK1 levels were downregulated under palmitic acid (PA) induction. Knockdown of cysteine-rich angiogenesis inducer (CCN1) could reduce the induction of EC inflammation and apoptosis by PA through inactivation of Wnt/β-catenin.^272^ Low stress activates Wnt/β-catenin and promotes endothelial mesenchymal transition (EndMT).^273^

Several studies suggest that Wnt1, Wnt2, and Wnt3a mediate a direct link between canonical Wnt signaling and VSMC proliferation in the neointimal formation of atherosclerosis.^274^ Upregulation of Wnt3a reduced the content of blood lipid, decreased the levels of inflammatory cytokines and oxidative stress, and increased plaque stability in ApoE^−/−^ mice.^275^ Canonical Wnt signaling and LRP5 are also involved in the formation of macrophage foam cells in humans.^276^

#### Potential therapeutic targets

Sclerostin, an inhibitor of canonical Wnt pathway, has been identified to prevent atherosclerosis.^265^ Linking extracellular inhibitor of Wnt/β-catenin signaling sclerostin and DKK1 (Dickkopf-1) to carotid intima-media thickness is recently reported in heart failure with reduced ejection fraction. Furthermore, DKK2 silencing alleviated M1 but increased M2 macrophage expression, and protected against lipid loading by activation of Wnt/β-catenin signaling.^271^ Matrix protein R-spondin 2 inhibits activation of the canonical Wnt/β-catenin pathway and lymphangiogenesis, and the resulting suppression of cholesterol efflux from atherosclerotic arteries,^277^ suggesting a novel role of Wnt in arterial cholesterol efflux. A more recent study characterized a novel role of Wnt signaling in enucleated erythrocytes and uncovered that regulation of the red blood cell (RBC) cytoskeleton and erythrocyte survival was controlled by noncanonical Wnt signals.^278^ Treatment of erythrocytes with Wnt5 increased RBC survival and improved oxygen delivery while maintaining normal cell morphology.^278^ The ability of the Wnt pathway to increase RBC flexibility may prove essential in combatting hypoxia by ensuring that RBCs better traverse occluded and inflamed vessels and capillaries. Perhaps increased oxygen delivery through improved erythrocyte health may slow the development of unstable plaque formation or thrombotic release and reduce the risk of myocardial infarction or stroke in patients with advanced atherosclerosis.^279^

## Atherosclerosis therapies

In view of atherosclerosis as the leading cause of death worldwide, prospective prevention and prompt treatment of atherosclerosis reduce the risk of developing its clinical manifestations. One of the critical causes of atherosclerosis is dyslipidemia. A number of therapeutic measures can reduce lipid risk factors for atherosclerosis. However, even when dyslipidemia is well controlled, risk factors from other sources remain. Therefore, it is required to address residual risk beyond lipids. Thus, we discuss the clinical trials that are currently underway for certain atherosclerotic processes, and the effects of quelling inflammation and atherosclerosis in the clinic (Table 1).

**Table 1:** Selected novel and emerging treatments for atherosclerosis

Type	Agent	Target	Mechanism	Phase	NCT number
Hypolipemiants	Alirocumab, evolocumab	PCSK9	Monoclonal antibody to PCSK9	Phase 3	NCT04790513
	Lerodalcibep	PCSK9	Anti-PCSK9 small binding protein	Phase 3	NCT04798430
	Inclisiran	PCSK9	Interfering RNA strategy targeting PCSK9	Phase 3	NCT04929249
	AT04A (AFF012)	PCSK9	Monoclonal antibody to PCSK9	Phase 1	NCT02508896
	Evinacumab	ANGPTL3	Monoclonal antibody to ANGPTL3	Phase 3	NCT04233918
	Angptl3 ASO	ANGPTL3	Antisense oligonucleotide targeting ANGPTL3	Phase 1	NCT02709850
	Bempedoic acid (ETC-1002)	ATP citrate lyase	Inhibitor of ATP citrate lyase	Phase 3	NCT03067441
	Lomitapide	MTP	An inhibitor of microsomal triglyceride transfer protein	Phase 3	NCT02145468
	Pradigastat (LCQ908)	DGAT1	A selective small-molecule DGAT1 inhibitor	Phase 2	NCT01474434
	Pelacarsen (TQJ230)	Lipoprotein(a)	Antisense oligonucleotides targeting lipoprotein(a)	Phase 3	NCT04023552
	CSL-112	ApoAI	A new formulation of apolipoprotein AI	Phase 3	NCT03473223
	Mipomersen	ApoB	Antisense oligonucleotide targeting apolipoprotein B100	Phase 3	NCT00794664
	Volanesorsen	ApoCIII	Antisense oligonucleotide targeting apolipoprotein CIII	Phase 3	NCT02658175
	Pemafibrate	PPARα	A selective PPARα modulator	Phase 3	NCT03071692
	ACP-501	Unknown	A recombinant human lecithin cholesterol acyltransferase	Phase 2	NCT03773172
	Gemcabene	ACC	Inhibitor of acetyl-CoA carboxylase	Phase 2	NCT02585869
	Icosapent ethyl	Unknown	A high-purity prescription form of eicosapentaenoic acid (EPA) ethyl ester	Phase 4	NCT04505098
Hypoglycemics	Lixisenatide (AVE0010)	GLP-1	Active receptor for endogenous incretin GLP-1	Phase 3	NCT01147250
	Liraglutide	GLP-1	GLP-1 receptor agonist	Phase 3	NCT04057261
	Semaglutide	GLP-1	GLP-1 analog	Phase 3	NCT05071417
	Exenatide	GLP-1	GLP-1 receptor agonist	Phase 2	NCT03287076
	Dulaglutide	GLP-1	Active GLP-1 receptor, increase cAMP in pancreatic islet β cell	Phase 3	NCT01394952
	Empagliflozin	SGLT-2	Inhibit the transporter SGLT-2 in glomerulus	Phase 4	NCT04461041
	Dapagliflozin	SGLT-2	Inhibit SGLT-2, increase natriuresis	Phase 3	NCT04564742
	Ertugliflozin	SGLT-2	SGLT-2 inhibitor	Phase 3	NCT03717194
	Canagliflozin	SGLT-2	Block reabsorption of glucose through kidney	Phase 3	NCT01032629
	Gliflozins	SGLT-2	Specific renal action by enhancing glucosuria	Phase 2	NCT04419337
	Vildagliptin	DPP-4	DPP-4 inhibitor	Phase 4	NCT01827280
	Linagliptin	DPP-4	Inhibiting the degradation of SDF-1α	Phase 4	NCT02350478
	Slogliptin	DPP-4	Enhance homing of endothelial progenitor cells	Phase 3	NCT00968708
	Saxagliptin	DPP-4	Catalyze GLP-1	Phase 4	NCT01552018
	Sitagliptin	DPP-4	DPP-4 inhibitor	Phase 2	NCT02576288

### Lipid-lowering drugs

#### PCSK9 inhibitors

As a serine protease, PCSK9 binds to the LDL receptor and targets it for lysosomal degradation, which provides an additional pathway to control plasma LDL cholesterol levels. Therefore, PCSK9 inhibitors are new and highly effective functions in lowering lipids.^280^ Although statins have been widely used, PCSK9 inhibitors are still needed to be used in patients with a high level of cholesterol.^281^ Because a majority large number of patients receiving statins or in combination with ezetimibe still fail to achieve their therapeutic goals.^282^ This treatment dilemma is actually exacerbated by the increasing demands on LDL levels in patients with cardiovascular disease.^280^

Targeting PCSK9 is at least one step ahead of statins for early intervention in lipid metabolism. Especially if patients can not tolerate statins due to adverse side effects, PCSK9 inhibitors are an alternative. In addition, PCSK9 inhibitors have been confirmed to be effective and safe in the treatment of familial hypercholesterinaemia.^283^ In recent meta-analyses, PCSK9 inhibitors such as two fully human anti-PCSK9 mAbs (alirocumab, evolocumab) showed a significant reduction in cardiovascular events such as coronary revascularization, myocardial infarction, and ischemic stroke, but fail to exhibit a beneficial effect on cardiovascular mortality.^284,285^ Currently, lerodalcibep using a PCSK9-binding domain (adnectin) and human serum albumin forming a recombinant fusion protein is another protein-based strategy to inhibit PCSK9 and has shown promising efficacy in phase III testing. In recent years, new approaches to block PCSK9 synthesis include inclisiran, a double-stranded siRNA that specifically targets and induces PCSK9 mRNA degradation, inhibits translation, protein synthesis, and plasma levels of PCSK9, thereby reducing the LDL-C levels in plasma.^286^ In clinical trials, inclisiran treatment results in a reduction by ~50% in plasma LDL-C levels.^287^ In addition, anti-PCSK9 vaccines are much less expensive to produce than anti-PCSK9 antibodies or siRNAs that require regular dosing. An anti-PCSK9 vaccine called AT04A consists of homologous mouse and mature human PCSK9 protein N-terminal epitope peptides from amino acid residues 153–692, which is able to reduce plasma lipid levels and hinder the development of atherosclerosis.^288^ Phase I clinical trial has demonstrated that AT04A is safe and immunogenic and exhibits significant LDL-C-lowering activity, justifying further development.^289^

#### ANGPTL3 inhibitors

The liver-specific secretory protein ANGPTL3 (angiopoietin-like proteins 3) significantly decreases in atherosclerotic cardiovascular disease. Loss of function of ANGPTL3 related to the benefits of lipid-lowering makes ANGPTL3 be a very attractive pharmacological target. ANGPTL3 inhibits the activity of endothelial lipase and lipoproteins lipase (LPL), resulting in elevated LDL and triglyceride (TG).^290^ Currently, two mechanisms have been found to affect ANGPTL3. A mAb targeting ANGPTL3 called evinacumab was designed to decrease LDL-C, non-HDL-C, and TGs by increasing the activity of LPL and other associated metabolic enzymes. In patients with homozygous familial hypercholesterinaemia, evinocumab could reduce the level of plasma LDL from 20% to 90% and TG ~50%.^291^ On the other hand, ANGPTL3-specific antisense oligonucleotides (ASOs) could inhibit ANGPTL3 synthesis. In the healthy group, this ASO could dose-dependently decrease TG by ~30% to 60%.^292^ In addition, LDL and HDL were highly positively affected. Future, clinical trials are needed to further quantify the effects of ANGPTL3 inhibitors on atherosclerotic cardiovascular disease.

#### ACL inhibitor

ATP citrate lyase (ACL) belongs to a type of cytosolic enzyme that works upstream of HMG-CoA reductase.^293^ Bempedoic acid is a recently approved oral compound that inhibits cholesterol synthesis pathways like statins, resulting in an increase in LDL receptor density and decreased cholesterol synthesis by inhibiting ACL. ACL links carbohydrates energy metabolism to fatty acids production by catalyzing the synthesis of acetyl-CoA, the essential substrate of fatty acids and cholesterol.^294^ In a large phase III trial, except for the same effect as statins or ezetimibe, bempedoic acid also exhibits a significant effect on LDL lowering with no increase in adverse events compared with placebo.^295–297^ However, further clinical trials on hard endpoints such as cardiovascular mortality and ischemic events are still lacking to date.

#### MTP inhibitor

Microsomal triglyceride transfer protein (MTP) takes functions as a stimulator of VLDL particle assembly and secretion through transferring TGs to ApoB. Lomitapide, an MTP inhibitor that blocks the assembly of metabolic precursors of LDL particles, is approved to treat familial hypercholesterolemia. As VLDL eventually changes to form LDL over time, reducing VLDL production while also decreasing the levels of LDL-C.^298^ Though previous clinical studies of lomitapide suggested a high incidence of gastrointestinal distress, transaminases, and fatty liver, these complications were offset by a decreased risk of atherosclerotic cardiovascular disease and improved quality of life.^299^ However, the follow-up clinical studies determined that lomitapide not only reduced the level of LDL-C, but also resulted in a reduction in plasma HDL-C and ApoAI.^300,301^ Owing to the combined benefits and risks, lomitapide is currently only available as an adjunctive orphan therapy to treat homozygous familial hypercholesterolemia.

#### DGAT1 inhibitor

Diacylglycerol acyltransferase 1 (DGAT1) takes a pivotal function in lipid metabolism by catalyzing the last step in the TG synthesis pathway. Pradigastat, an orally administered DGAT1 inhibitor is another treatment modality. In phase I/II trials, six familial chylomicronaemia syndrome patients showed a dose-dependent reduction in TG of 41–70% over 21 days of treatment with only mild transient gastrointestinal adverse events in patients receiving dose-ranging pradigastat.^302^ A trial from 106 participants with overweight or obesity receiving multiple escalating doses, pradigastat decreased postprandial glucose, insulin, and TG excursions while increasing postprandial glucagon-like peptide-1 (GLP-1) levels.^303^ Gastrointestinal adverse reactions, including nausea and diarrhea, are significant at the dose of 10 mg; dietary fat reduction improves tolerability, but these still limit the development and widespread use of this drug.^303^

#### Apo inhibitors and mimetic peptides

Lipoprotein(a) [Lp(a)] composed of ApoB100 covalently bound to Apo(a), has been recognized as an independent risk factor and a pro-atherogenic potential for cardiovascular disease, while low Lp(a) levels are commonly associated with a reduced risk of cardiovascular disease.^304^ Preclinical studies established that ASO IONIS-APO(a)_RX_ specifically targeted hepatic LPA mRNA to reduce the levels of plasma Lp(a).^305^ Similar to IONIS-APO(a)_RX_, the research group later developed IONIS-APO(a)-L_Rx_ (now called AKCEA-APO(a)-L_Rx_) that exhibited specific and efficient targeting to the liver. In phase I/II clinical study, IONIS-APO(a)-L_Rx_ have exhibited a prominent dose-dependent decrease in mean levels of Lp(a) without major adverse events and other clinical findings. Recently, pelacarsen (also termed AKCEA-APO(a)-L_Rx_ or TQJ230), is undergoing a phase III clinical study that is designed to recruit at least 7500 patients with a history of symptomatic peripheral arterial disease, ischemic stroke, or myocardial infarction (NCT04023552).

Mipomersen is a novel type of second-generation antisense oligonucleotide and commonly accumulates in hepatocytes wherein it binds to ApoB mRNA and limits mRNA availability, resulting in a decrease in the production of ApoB and other lipoproteins that need ApoB including LDL, VLDL, and chylomicrons.^298^ A meta-analysis demonstrated that, unlike lomitapide, mipomersen could significantly reduce Lp(a) by 20–40%, non-HDL-C by 28%, LDL by 35–47% without influencing the level of HDL-C.^306^ Though numbers of clinical trials have suggested that mipomersen has efficacy in lowering ApoB-related lipoproteins in patients with different hypercholesterolemic phenotype, its approval is currently only available to treat homozygous familial hypercholesterolemia as an adjunctive orphan drug due to adverse effects such as flu-like symptoms and transaminitis.^306–308^

Similar to mipomersen, as a second-generation chimeric antisense inhibitor for ApoCIII, volanesorsen blocks protein synthesis as well as limits mRNA availability through binding to the ApoCIII mRNA. ApoCIII inhibits hepatic uptake of LPL and TG-rich particles, which have been recognized as high risks to result in hypertriglyceridemia.^309^ Volanesorsen Phase II study showed a significant reduction in TGs ~40–80% compared with placebo, either as monotherapy or in combination with fibrates/statins.^310^ Currently, volanersorsen has completed a phase III clinical trial for patients with familial chylomicronemia syndrome (NCT02658175). Considering that TG and ApoCIII are important factors inducing arteriosclerotic cardiovascular disease, volanesorsen is expected to improve overall cardiovascular outcomes in the future.

CSL-112 is a superior type of rHDL infusion therapy containing purified native human ApoAI from human plasma and phosphatidylcholine derived from soybeans.^311^ Numerous phase I/II clinical studies have determined that CSL-112 is able to increase ApoAI, pre-β HDL levels, cholesterol efflux capacity with good tolerance, with no evidence of liver toxicity.^312–314^ At present, CSL-112 is being studied in phase III clinical trial that is aimed to enroll not <17,000 volunteers to assess the endpoint of major adverse cardiovascular effects (MACEs) in adults having a history of acute myocardial infarction (NCT03473223).

#### ACP-501

Lecithin cholesterol acyltransferase (LCAT) is an enzyme in plasma that mainly catalyzes cholesterol esterification reaction during HDL formation. ACP-501 is a solution of recombinant human LCAT produced in CHO cells. It catalyzes free cholesterol to form cholesteryl esters with concomitant production of HDL-C.^315^ In a phase I clinical trial, ACP-501 was commonly associated with a dose-dependent increase in the level of HDL-C up to 42% and a decrease in cholesterol esters of ~22% in patients with low levels of HDL-C and atherosclerotic heart disease.^316^ The recent study involving patients with ST-elevation myocardial infarction has confirmed dose-related increases in HDL cholesterol esters, TC esters, and HDL-C in these patients.^317^ Although current clinical trial results of ACP-501 have shown benefits in LCAT deficient individuals, it still needs to demonstrate whether these benefits apply to other high-risk patients with dysfunctional and low HDL-C.

#### PPARα modulator

The PPARα agonists belong to ligand-activated transcriptional regulators that are involved in regulating lipid metabolism and expression of key apoproteins that influence the level of HDL and TG-rich lipoproteins, control cholesterol transport in macrophages and affect inflammation response.^318^ As a novel and specific PPARα modulator, Pemafibrate can maximize the beneficial effects and minimize the adverse effects of fibrates used currently. In vivo and in vitro experiments have confirmed that pemafibrate has a more potent effect on PPARα activation than previous fenofibrate. Recently, a randomized and double-blind phase II clinical study has demonstrated that pemafibrate has a greater effect on lowering TG and HDL-C levels than fenofibrate.^319^ Moreover, the incidence of adverse effects is lower than that of fenofibrate.

#### Acetyl-CoA carboxylase inhibitor

Gemcbene, as an inhibitor of acetyl-CoA carboxylase (ACC), is a dicarboxylic acid, which can reduce the production of hepatic triglycerides and cholesterol and enhance the clearance of VLDL cholesterol.^320^ Phase II clinical trials demonstrated that compared with placebo gemcbene could significantly reduce plasma LDL-C, VLDL-C, ApoCIII, TG, and CRP levels and obviously increased HDL-C levels.^321^ Gemcbene will now continue its phase II POC trial and assess the “End of Trial” outcomes jointly with the US Food and Drug Administration to plan the anticipated phase III clinical trial. Currently, as for patients with homozygous familial hypercholesterolemia, gemcabine may be a potentially valuable therapy because of lowering LDL-C independent of LDLR function.^322^

#### Icosapent ethyl

Icosapent ethyl produced by the esterification of high-purity eicosapentanoic acid, has been approved for treating severe hypertriglyceridemia and reducing the risk of cardiovascular disease in patients with high TG levels and pre-existing atherosclerotic cardiovascular disease exceeding maximum intensity statin treatment.^323^ Recent clinical trials suggested a reduction by approximately 25% in cardiovascular disease events, and an obvious return in low attenuation plaque volume.^324^ Therefore, icosapent ethyl may be a good addition for patients with elevated TG levels to reduce LDL-C.

### Glucose-lowering drugs

#### GLP-1 receptor agonists

Glucagon-like peptide-1 (GLP-1) has been found to show incretin-like activity, leading to enhanced glucose-induced insulin secretion in normal and diabetic humans. These findings and subsequent evidence suggest that GLP-1 inhibits gastric emptying, food intake, and glucagon secretion supporting to development GLP-1 receptor agonists (GLP-1 RA) for the treatment of type 2 diabetes mellitus (T2DM).^325^ GLP-1 RAs mainly include exenatide, liraglutide, albiglutide, dulaglutide, lixisenatide, benaglutide, and semaglutide. GLP-1 receptor agonists, such as exenatide and lixisenatide were based on exendin structure and artificially synthesized, which showed a low homology to human GLP-1 and also had a resistant effect on the degradation of dipeptidyl peptidase-4 (DPP-4).

Lixisenatide is the first GLP receptor agonists to undergo extensive cardiovascular studies. Clinical trials showed that lixisenatide did not obviously alter the incidence of major cardiovascular events in >6000 patients with T2DM at risk for atherosclerosis.^326^ Therefore, lixisenatide did not differ from the placebo group in the rate of cardiovascular diseases in T2DM patients with high cardiovascular risk. Although the results regarding cardiovascular benefits are neutral, lixisenatide should not be discarded. Because the associated glycemic reduction may also decrease the long-term risk of cardiovascular and microvascular disease.^327^

Another class of GLP-1 receptor agonists is based on the natural human GLP-1 structure, which is processed by local modification of human GLP-1 molecular structure and has high homology to human GLP-1amino-acid sequences, such as liraglutide and benaglutide. The occurrence of MACEs, cardiovascular disease mortality, and total mortality was decreased in liraglutide-treated subjects. The incidence of microvascular disease, primarily reflecting a decrease in microalbuminuria events, was also lower in the liraglutide arm.^328^ Semaglutide, a GLP-1 RA structurally similar to liraglutide, was found to reduce MACEs occurrence which mainly reflected a reduction in the numbers of non-fatal strokes and myocardial infarction. A recent study indicates that semaglutide is associated with persistent, clinically relevant weight loss.^329^

The dulaglutide and cardiovascular outcomes in T2DM study showed that dulaglutide reduced the composite cardiovascular outcomes during 5-year period. Furthermore, liraglutide can be added to the management of patients with diabetes and other risk factors of cardiovascular diseases to decrease glucose concentrations, minimize hypoglycemia, decrease blood pressure and weight, and decrease the risk of cardiovascular diseases. However, the rate of gastrointestinal adverse events was higher than in patients using dulaglutide than other GLP-1 RAs.^330^

#### SGLT-2 inhibitors

Selective sodium-glucose co-transporter 2 inhibitors (SGLT2i) provide an insulin-independent mechanism to decrease glucose levels and have been approved as a therapeutic strategy for T2DM. They promote urinary glucose excretion (up to ~50%) by decreasing the reabsorption of glucose in the urine by the proximal tubules of the kidneys.^331^ Currently, findings show that despite only a modest decrease in glycated hemoglobin, SGLT-2 inhibitors improve cardiovascular outcomes in T2DM patients, with no conclusive evidence of additional safety issues.^332^ Researches suggested that SGLT2i exhibited a moderate benefit to decrease the risk of MACE, which appeared to be limited to patients with confirmed atherosclerotic cardiovascular disease. But, regardless of a history of heart failure or atherosclerotic cardiovascular disease, they have substantial benefits in reducing heart failure hospitalizations and kidney disease progression.^333^

Empagliflozin, ertugliflozin, dapagliflozin and canagliflozin were all belongs to SGLT2i. Ertugliflozin was demonstrated the non-inferiority versus placebo on MACE in patients with atherosclerotic cardiovascular disease and T2DM.^334^ Dapagliflozin treatment did not lead to lower or higher MACE compared to placebo in T2DM patients at risk for atherosclerotic cardiovascular disease rate but did decrease the rate of heart failure or cardiovascular death hospitalizations.^335^ Like other drugs in its class, empagliflozin has a lower inherent risk of hypoglycemia due to its insulin-independent mechanism of action, allowing it to be used as monotherapy and in combination with other anti-diabetic drugs with complementary modalities components to improve glycemic control efficiency in T2DM patients. In addition to lowering glucose levels, empagliflozin has beneficial effects on many non-glycemic outcomes, including modest reductions in blood pressure and body weight. As an adjunctive treatment to standard of care, it demonstrated cardioprotective and renoprotective properties in a mandatory cardiovascular outcome trial that was largely independent of glycemic control in patients with established cardiovascular disease and T2DM. Given its apparent cardioprotective effect, this drug may be worthy of prioritization among patients at high risk for cardiovascular events who require additional anti-diabetic drugs to achieve glycemic goals.^336^ As an SGLT2i, canagliflozin, decreases blood pressure, body weight, glycemia, and albuminuria in patients with diabetes. In two trials involving T2DM patients at high risk of cardiovascular disease, patients receiving canagliflozin had a lower risk for cardiovascular events than those receiving placebo, but a higher risk of amputation.^337^

#### DPP-4 inhibitors

Gliptins belong to a large class of anti-diabetic drugs inhibiting the activity of DPP-4, which is the major enzyme responsible for the degradation of GLP-1, thereby lowering blood glucose.^338^ Clinical trials suggested that DPP-4 inhibition could help hinder a variety of factors that lead to the development and progression of atherosclerosis, such as lowering lipid levels in plasma, inhibiting inflammatory response, and promoting vasodilation.^339^ In addition, the risk of hypoglycemia was lower when DPP-4 inhibitors were co-administered with sulfonylureas.^340^ Hypoglycemic episodes, especially severe episodes of hypoglycemia, are accompanied by an increased risk of major cardiovascular events and mortality in T2DM people.^341^ DPP-4 inhibitors, such as alogliptin, linagliptin, saxagliptin, sitagliptin, and vildagliptin have been widely available globally, which show a similar effect on reducing the risk of MACE. DPP-4 inhibition neither increases nor decreases the risk of the composite MACE outcome, including acute myocardial infarction, ischemic stroke, or cardiovascular death, with or without hospitalization for unstable angina in the composite endpoint. Therefore, the cardiovascular safety of these drugs has been demonstrated.^341^

The glycemic control of DPP-4 inhibitors varies by molecule, with monotherapy reducing glycated hemoglobin (HbA1c) by an average of 0.5–1.0%. Combination therapy with other anti-diabetic drugs may result in additional effects of HbA1c reduction.^342^ Alogliptin, linagliptin, saxagliptin, and sitagliptin have undergone a number of rigorous clinical studies in recent years, and the data of vildagliptin on safety and tolerability are available from various studies in meta-analyses obtained from a retrospective analysis.^342^

Linagliptin is one of the latest DPP-4 inhibitors in the gliptins market because it offers cardiovascular protection and renal safety.^343–345^ A large-scale clinical trial of linagliptin versus glimepiride on MACEs in T2DM patients concluded that among people with relatively early T2DM and elevated the risk of cardiovascular disease, receiving linagliptin led to a non-inferior risk for the composite cardiovascular outcome.^346^ Meanwhile, as for T2DM patients with renal and high cardiovascular risk, the addition of linagliptin to standard care led to a non-inferior risk for the composite cardiovascular outcome compared with placebo.^347^

The cardiovascular safety of sitagliptin was validated in trials evaluating cardiovascular outcomes with sitagliptin.^348^ Report showed that the risk of clinical adverse events did not increase during 18 weeks of sitagliptin treatment in patients in China, India, and South Korea.^349^ Notably, the major risk of cardiovascular events and hospitalization for heart failure have been assessed in diverse studies. Sitagliptin monotherapy did not increase morbidity/mortality and the risk of macrovascular/microvascular complications in T2DM.^350^ Meanwhile, long-term effects of sitagliptin on hospitalizations for heart failure were proven safe. A 3-year follow-up clinical study showed that sitagliptin did not increase the risk of hospitalization for heart failure or cardiovascular diseases.^348^ In addition, the incidence of in-hospital reinfarction, acute renal failure, and pulmonary edema were obviously decreased in patients with acute coronary syndrome and diabetes using sitagliptin.^351,352^ Sitagliptin showed even better results in reducing the rate of major cardiovascular events in patients with diabetes.^353^ Overall, sitagliptin is well tolerated as monotherapy or in combination with other common anti-diabetic drugs.

### Targeting inflammatory pathways

Current treatments for atherosclerosis mainly focus on modulating traditional risk factors, including lipid-lowering and glucose-lowering drugs, to prevent and delay plaque formation and progression. Therapeutic drugs mainly include statins and anti-diabetic agents as well as metformin, which also exert anti-inflammatory effects. Anti-inflammatory interventions may represent novel potential therapies with additional beneficial effects in high-risk patients. We summarize the main therapeutic strategies in the following sections (Table 2).

**Table 2:** Overview of clinical studies targeting inflammatory pathways in atherosclerosis

Agent	Target	Mechanism	Phase	NCT number
Canakinumab (ACZ885)	IL-1β	Monoclonal antibody targeting IL-1β	Phase 3	NCT01327846
Anakinra	IL-1R	IL-1R blocker	Phase 2/3	NCT01950299
Proleukin	IL-2	Recombinant human IL-2	Phase 1/2	NCT03113773
Methotrexate	Unknown	Inhibits inflammatory cytokines production	Phase 2/3	NCT00759811
Ziltivekimab	IL-6	Monoclonal antibody targeting IL-6	Phase 3	NCT05021835
Tocilizumab	IL-6R	Monoclonal antibody against IL-6 receptor	Phase 3	NCT04888221
Sarilumab	IL-6R	IL-6 receptor blocker	Phase 4	NCT04350216
Etanercept	TNF	A dimeric fusion protein that inhibits the binding of both TNF-α and TNF-β to TNF receptors	Phase 4	NCT02109289
Dapansutrile (OLT1177)	NLRP3	NLRP3 inflammasome inhibitor	Phase 1	NCT03534297
Colchicine (LoDoCo2)	NLRP3	NLRP3 inflammasome inhibitor	Phase 4	NCT05130892
Losmapimod	p38 MAPK	A selective inhibitor of p38 MAPK	Phase 3	NCT02145468
Inclacumab	P-selectin	Monoclonal antibody targeting P-selectin	Phase 3	NCT04927247
Darapladib (SB-480848)	Lp-PLA_2_	An inhibitor of Lp-PLA2	Phase 3	NCT00799903
Varespladib (LY315920)	sPLA_2_	a selective sPLA2 inhibitor	Phase 3	NCT01130246
Metformin	Unknown	Limits oxidative stress by inhibiting mitochondrial complex I	Phase 2	NCT03772964
Succinobucol (AGI-1067)	Unknown	An oxidative-inflammatory inhibitor	Phase 3	NCT00066898

#### Interleukin inhibitors

Experimental data on animals susceptible to atherosclerosis have confirmed that either pharmacological inhibition or genetic deletion of IL-1 signal pathways decreases atherosclerotic plaque area and progression rates. On the contrary, either increasing active IL-1 via injection of exogenous IL-1β, or reducing IL-1Ra, can result in plaque growth and exacerbation of atherosclerosis.^354^ Based on the above findings, the anti-inflammatory antithrombotic outcomes study of canakinumab aimed to demonstrate the role of the IL-1 signal pathway in atherosclerosis. Canakinumab (300 mg four times yearly) decreased the rates of recurrent MACE such as cardiovascular death, myocardial infarction, and ischemic stroke.^355^ Subsequent large-scale clinical trials firstly confirmed the efficacy of anti-inflammatory interventions in patients at risk for atherosclerotic events. The individual participants treated with canakinumab had a small but statistically significant increase in the risk of infection containing those with fatal outcomes. Furthermore, interventional treatments with canakinumab and IL-1Ra agonist (anakinra) demonstrated a key pathological role of IL-1β in the induction of inflammation-related diseases.^356^ These findings, therefore, provided evidence that inhibition of IL-1β could ameliorate the clinical outcome of cardiovascular disease.

The pleiotropic cytokine IL-6 plays a key role in numerous pathological processes, such as atherosclerosis and rheumatic diseases, which exhibits both pro- and anti-inflammatory properties depending on the targeted cell type. Ziltivekimab is a novel human IgG1, κ antibody directed against IL-6 ligand that has been previously investigated to examine the safety and pharmacodynamic effects in phase I/II trials of patients with rheumatoid arthritis (NCT01559103) and chronic kidney disease (NCT03126318). A recent study supported that ziltivekimab could cause a decrease in overall cardiovascular risk in patients with increased inflammation on hemodialysis by reducing inflammation.^357^

Tocilizumab and sarilumab, both mAbs against IL-6R, have been approved for combination used with other types of antirheumatic agents or as monotherapy in patients with rheumatoid arthritis and other inflammation-related diseases.^358^ Tocilizumab and sarilumab were tested in clinical trials for their efficacy as rheumatoid arthritis treatments among patients with cardiovascular disease.^359^ Though tocilizumab significantly perturbs cholesterol and lipid homeostasis, including increasing TC and LDL, it also shows beneficial effects on surrogate markers of cardiovascular risk. Other clinical studies of large numbers of patients with rheumatoid arthritis using tocilizumab in the postmarketing setting exhibit a lower rate of MACE.^360^

Methotrexate and folic acid are analogs of similar chemical structures. Preclinical studies have confirmed that methotrexate inhibits lymphocyte proliferation and inflammatory cytokines production via adenosine binding with A_2_ receptor, which is one of the most important anti-inflammatory mechanisms.^361^ Previous systematic reviews and meta-analyses suggested the significant relationships between methotrexate treatment and a reduced risk of cardiovascular disease.^362^ Similarly, a recent study of patients with rheumatoid arthritis showed that methotrexate use is associated with lower cardiovascular outcomes.^363^

#### NLRP3 inflammasome inhibitors

The NLRP3 inflammasome takes an important function in proteolysis and IL-1β maturation. As an essential sensor, functions in pathological processes are related to vascular endothelial dysfunction.^364^ Thus, targeting NLRP3 may be an effective and potential target for therapeutic intervention in acute and chronic cardiovascular disease. Colchicine, a natural product extracted from the bulb of autumn colchicum, has been successfully used in the treatment of inflammatory diseases for millennia. In the field of cardiovascular research, a number of studies have determined the effect of colchicine in decreasing recurrent cardiovascular events in atherosclerotic patients.^365^ Likewise, the low-dose colchicine trial-2 trial in patients with the chronic phase of coronary artery disease suggested similar efficacy.^366^

Dapansutrile, a specific NLRP3 inflammasome inhibitor, phase I randomized trial has demonstrated its safety and tolerability in heart failure patients with reduced left ventricular ejection fraction.^367^ According to this preliminary data, a dose of 2000 mg/d dapansutrile appears to be the possible dose to be tested in future studies. However, dapanusutrile appears no significant effect on C-reactive protein levels, the preferred inflammatory biomarker for cardiovascular risk stratification, and IL-1 itself is downstream of the activated NLRP3 inflammasome.^368^

#### p38 MAPK inhibitor

p38 MAPK is activated by multiple factors in the cardiovascular system, including oxLDL, hypertension, ischemia, and volume overload.^369^ Losmapimod is an oral, selective, and reversible competitive p38 inhibitor targeting MAPK activation. Early studies indicated the pharmacodynamic and pharmacokinetic basis of losmapimod in healthy and positive data on the safety and tolerability of losmapimod. However, in the phase III trial, using losmapimod treated acute myocardial infarction patients did not show a relationship with a decreased risk of the MACE.^370^ Notably, a disadvantage of the trial was the limited treatment course, whereby we could not exclude the possibility that lomatimod may be effective as an anti-inflammatory treatment for a longer duration.^371^

#### P-selectin antagonist

Inclacumab directly targets the cell adhesion molecule P-selectin, taking functions at the interface of thrombosis and inflammation. Inclacumab has appeared to significantly reduce myocardial damage in certain types of acute and chronic myocardial infarction.^372^ In addition, the P-selectin has been identified as the independent risk factor for peripheral arterial disease and decreased ankle-brachial index.^373^ Though the basic principle indicates that P-selectin antagonist inclacumab is able to improve the development of peripheral artery disease, evidence from clinical studies is lacking.^374^

#### Lp-PLA2 and sPLA2 inhibitors

The level and activity of lipoprotein-associated phospholipase A2 (Lp-PLA2) and secretory phospholipase A2 (sPLA2) in circulating blood have been confirmed to be biomarkers of increased risk of atherosclerotic cardiovascular disease.^375^ Two potent inhibitors against PLA2, darapladib (Lp-PLA2 inhibitor) and varespladib (sPLA2 inhibitor), have been investigated extensively in clinical studies. Phase III clinical trial on darapladib against ischemic events in stable coronary heart diseases showed that darapladib could not obviously decrease the risk of the primary composite endpoint of myocardial infarction, stroke, or cardiovascular death (NCT00799903). Similarly, a double-blind, randomized, multicenter trial also suggested that varespladib could not decrease the risk of recurrent cardiovascular events, but obviously increased the risk of myocardial infarction (NCT01130246). These findings indicate that besides serving as an inflammatory biomarker of cardiovascular disease deeper knowledge on PLA2 is needed.^376^

#### Succinobucol

Succinobucol is a novel type of phenolic antioxidant small molecule that is not easily degraded or modified by body metabolism, thereby inhibiting the formation of the hepatotoxic molecule spiroquinone and exhibiting an anti-inflammatory effect by binding to VCAM-1.^377^ In the phase III trial, 6144 patients at risk of cardiovascular disease were randomly divided into succinobucol and placebo group. Regretfully, the primary composite endpoint of myocardial infarction, unstable angina, cardiovascular death, resuscitated cardiac arrest, coronary revascularization or stroke did not decrease. However, the risk of key secondary endpoint was decreased by 19%, and patients’ risk of new-onset diabetes was decreased by 64%. Based on these, further studies may be required to elucidate the clinical effect of succinobucol on cardiovascular events in the future.

## Conclusion and remarks

Atherosclerosis is a chronic inflammatory vascular disease primarily triggered by vascular cells and immune cells. In recent decades, inflammatory mediators, as potential common risk factors, have received extensive attention, which influences LDL and triglyceride-rich lipoprotein levels, and alters artery wall cell behaviors to beckon leukocytes. Rather than replacing traditional drivers like LDL, the concept of inflammation as a driver of atherosclerosis provides more mechanisms by which the traditional risk factors induce atherosclerotic disease and its complication. The development of single-cell analysis technologies provides deeper insights into the roles of various cells involved in atherosclerotic lesions. The contribution of VSMCs to atherosclerotic plaque development is largely underestimated previously.^1,33^ Advances in lineage tracing provide an available access route to re-evaluate this misdiagnosing situation. However, many questions still remain to be answered. For example, what are the roles of different VSMC phenotypes; how are the processes of VSMC phenotypic switching and transdifferentiation to other type-like phenotypes such as macrophage-like or myofibroblast-like connected. Though we understand some of the molecular mechanisms that regulate phenotypic switching of VSMCs, and a positive, negative or neutral effect of particular VSMC phenotypes on atherosclerosis and plaque stability, we currently lack experimental evidence in vivo from animal research or correlated data in human studies. Various immune cells are accumulated within complicated plaques, propagating the development of atherosclerosis and its complications. Recent reports highlight the complexity of the origins of lesion macrophages as well as the plasticity of these cells and uncover the specific characteristics of immune cell dysregulation within atherosclerotic plaques that give rise to clinical cardiovascular events, representing that identification of specific dysregulations of immune cells in the plaques associated with cardiovascular events may be required for the development of more precise immunotherapies of atherosclerosis.

A growing body of evidence demonstrates that the NLRP3 inflammasome and TLRs contribute to the development of atherosclerosis, and are identified to be a promising new target for the treatment of these diseases. Given the intimate link between dysregulation of NLRP3 inflammasome and atherosclerosis, it is both challenging and intriguing to reveal the complex mechanisms of regulation and activation of NLRP3 inflammasome and their influence on human health and disease. In addition, studies on the role of PCSK9 in different cells involved in at all stages of the plaque have supported that the effect of PCSK9 on atherosclerotic development is beyond the LDLR degradation, and occurs by crosstalking with other molecular mechanisms ranging from the activation of inflammatory pathways to apoptosis of ECs and migration of VSMCs. Therefore, it is needed to deeply understand the roles of PCSK9 in atherosclerosis by unveiling the precise molecular mechanisms underlying it.

Inflammation has opened the door to more new therapeutic targets for atherosclerosis treatment. In the last years, three clinical trials have translated this large body of genetic evidence and human biomarker and experimental work to clinical fruition.^355,366,378^ Indeed, the recent evidence that interventions of anti-inflammation can forestall the complications of atherosclerosis scratches merely the surface of the potential as novel therapeutics. NLRP3 inflammasome and the downstream pro-inflammatory cytokines IL-1β and IL-18 are attractive pharmaceutical candidates for therapeutic intervention of atherosclerosis, and selectively neutralizing these cytokines has been shown to be available in the context. Macrophage-targeting NPs can deliver a broad range of therapeutic drugs to atherosclerotic lesions, which suppresses pro-atherogenic processes of macrophages, promoting inflammation resolution and plaques stabilization.^71^ The challenge facing us is how to enhance beneficial efficiency, as interference with inflammatory signalings can impair host defenses. It is likely that the advances in multiple omics technologies will help to solve the multidimensional problem caused by the plentiful number of mediators and myriad of pathways that are susceptible to therapeutic intervention. Although translating basic findings to clinical treatment is challenging, increasing understanding of inflammation in atherosclerosis facilitated by recent technical advances provides optimism. Therefore, further work remains to be performed in harnessing the inflammatory concept to ameliorate and forestall atherosclerosis and its complications. Many unanswered questions in this field provide opportunities for future research and may yield therapeutic strategies to improve patient outcomes.
